# Estimating Finite Rate of Population Increase for Sharks Based on Vital Parameters

**DOI:** 10.1371/journal.pone.0143008

**Published:** 2015-11-17

**Authors:** Kwang-Ming Liu, Chien-Pang Chin, Chun-Hui Chen, Jui-Han Chang

**Affiliations:** 1 Institute of Marine Affairs and Resource Management, National Taiwan Ocean University, Keelung 202, Taiwan; 2 George Chen Shark Research Center, National Taiwan Ocean University, 2 Pei-Ning Road, Keelung 202, Taiwan; 3 Center of Excellence for the Oceans, National Taiwan Ocean University, 2 Pei-Ning Road, Keelung 202, Taiwan; 4 Institute of Oceanography, Nation Taiwan University, Taipei 106, Taiwan; 5 Fisheries Research Institute, Council of Agriculture, 199, Heyi Road, Keelung 202, Taiwan; 6 School of Marine Sciences, University of Maine, Orono, Maine 04469, United States of America; Hellenic Centre for Marine Research, GREECE

## Abstract

The vital parameter data for 62 stocks, covering 38 species, collected from the literature, including parameters of age, growth, and reproduction, were log-transformed and analyzed using multivariate analyses. Three groups were identified and empirical equations were developed for each to describe the relationships between the predicted finite rates of population increase (λ’) and the vital parameters, maximum age (T_max_), age at maturity (T_m_), annual fecundity (f/R_c_)), size at birth (L_b_), size at maturity (L_m_), and asymptotic length (L_∞_). Group (1) included species with slow growth rates (0.034 yr^-1^ < k < 0.103 yr^-1^) and extended longevity (26 yr < T_max_ < 81 yr), e.g., shortfin mako *Isurus oxyrinchus*, dusky shark *Carcharhinus obscurus*, etc.; Group (2) included species with fast growth rates (0.103 yr^-1^ < k < 0.358 yr^-1^) and short longevity (9 yr < T_max_ < 26 yr), e.g., starspotted smoothhound *Mustelus manazo*, gray smoothhound *M*. *californicus*, etc.; Group (3) included late maturing species (L_m_/L_∞_ ≧ 0.75) with moderate longevity (T_max_ < 29 yr), e.g., pelagic thresher *Alopias pelagicus*, sevengill shark *Notorynchus cepedianus*. The empirical equation for all data pooled was also developed. The λ’ values estimated by these empirical equations showed good agreement with those calculated using conventional demographic analysis. The predictability was further validated by an independent data set of three species. The empirical equations developed in this study not only reduce the uncertainties in estimation but also account for the difference in life history among groups. This method therefore provides an efficient and effective approach to the implementation of precautionary shark management measures.

## Introduction

Sharks are the top predators in the ocean and play an important role in the marine ecosystem [1, 2]. Recent estimates indicated that shark populations have declined significantly in many regions of the world [3, 4, 5, 6, 7]. Worldwide trade in shark fin has increased dramatically. In 1980, the figure was less than 2000 MT, but by 2000 this had risen to 11602 MT [8], indicating a significant increase in shark exploitation during that period, but the shark landings deceased thereafter [9]. As a result, shark conservation and management have become issues of great concern in recent years. Many countries and international management and conservation organizations have taken their own steps with respect to sharks. For example, the USA, Australia, and the Maldives have regulations controlling the total allowable catch (TAC) and have also limited fishing grounds. According to the International Union for the Conservation of Nature and Natural Resources (IUCN) red list criteria, 32% of open ocean sharks are now considered threatened [9]. The convention on International Trade in Endangered Species of Wild Fauna and Flora (CITES) has placed the whale shark, *Rhincondon typus*, basking shark, *Cetorhinus maximus*, great white shark, *Carcharodon carcharias*, scallooped hammerhead, *Sphyrna lewini*, smooth hammerhead, *S*. *zyganea*, great hammerhead, *S*. *mokarran*, oceanic whitetip, *Carcharhinus longimanus*, porbeagle shark, *Lamna nasus* and manta rays, *Manta* spp. on its Appendix II list [10]. All these various measures serve to accentuate the urgency of shark management. Consequently, the regional fisheries management organizations have taken various management measures for sharks, i.e. prohibition of bigeye thresher, *Alopias superciliosus*, silky, *Carcharhinus falciformis*, oceanic whitetip, and Sphyrnidae except for *Spyrna tiburo* retaining on board in the Atlantic Ocean [11], prohibition of oceanic whitetip and thresher sharks, *Alopias* spp. retaining on board in the Indian Ocean [12], and prohibition of oceanic whitetip and silky shark retaining on board in the Pacific Ocean [13].

At least 498 species (8 orders) of sharks exist worldwide [14]. Many different life history traits have been found among these species. Maximum size ranges from 22 cm total length (TL) for the spined pigmy shark, *Squaliolus laticaudus* [15] to 1800 cm TL for the whale shark, *Rhincodon typus* [16]. Growth rates range from k = 0.034 yr^-1^ for the pike dogfish, *Squalus acanthias* [17] to k = 0.358 yr^-1^ for the spadenose shark, *Scoliodon laticaudus* [18]. In terms of reproductive strategy, three general categories have been identified: oviparity, viviparity, and aplacental viviparity. However, the litter size varies remarkably among species even for those falling within the same reproductive type. For example, for viviparous sharks, litter size ranges from six for the basking shark [19] to 82 for the blue shark, *Prionace glauca* [20]. For aplaental viviparous sharks, litter size ranges from two for the bigeye and pelagic thresher shark [21, 22] to more than 300 for the whale shark [23] (S1 Table). It is clear that, compared to teleosts, sharks have a far more complex and varied set of life history traits particularly the reproductive traits.

Due to the fact that sharks have a much lower commercial value than tunas and other teleost fish, catch, effort, and bycatch data for shark species are not readily available. Consequently, conventional stock assessment methods, such as surplus production and stock-recruitment models, have seldom been applied to examine shark population dynamics despite of recent works on blue sharks [24, 25]. However, because sharks have similar life histories to mammals, demographic models which have been applied to mammals have been found to better describe the dynamics of shark populations [26].

To date, the assessment of shark stock status using demographic analysis has, for the most part, been based on the hypothesis of a unit stock [26, 27, 28, 29, 30, 31, 32, 33, 34, 35, 36, 37]. However, this approach needs detailed information on vital parameters such as natural mortality, age at maturity, litter size, reproductive cycle, and longevity. It is difficult to apply this approach to species with limited available life history information [38], and only few demographic models consider density-dependent effects. There is an urgent need to manage and conserve shark stocks, and empirical equations based on vital parameters, which could be used to estimate the finite rate of population increase for particular categories of shark would make this task easier and more efficient based on a precautionary approach.

A number of authors have applied multivariate analyses, including principal component analysis (PCA), cluster analysis (CA), and regression analysis, to fish resource management [38, 39, 40, 41, 42]. Using PCA, Winemiller and Rose [39] identified four categories of species, namely periodical, opportunist, equilibrium, and intermediate. They suggested using size limits and maintaining adult abundance to manage and protect teleost fish larger than 100 cm and large sharks. King and McFarlane [38] also used PCA to identify three groups based on the vital parameters of growth rate, litter size, asymptotic length, and size at birth. They concluded that stock assessment is required every 1–2 years for species with a short life span, fast growth and small litter size. Cortés [41] applied CA to shark data and identified three groups based on litter size, longevity, asymptotic length, size at birth, and growth rate. Jennings et al. [40] used regression analysis to estimate vital parameters and predict fish abundance. Frisk et al. [43] described the effects of size at maturity and age at maturity on maximum observed length for elasmobranches. However, none of these studies has provided an empirical equation to estimate the finite rate of population increase.

The objectives of this study were 1) to use multivariate analysis to categorize sharks into groups based on their vital parameters, 2) to develop an empirical equation to estimate the finite rate of population increase for each group, and 3) to propose appropriate management measures for each group. It is hoped that these empirical equations can be applied to other shark species with limited life history information so as to achieve the goal of precautionary management.

## Materials and Methods

In our search of the existing literature, we collected and analyzed vital parameter data from 83 studies. Only stocks with complete data (both age and growth and reproduction) were analyzed. In total, data of vital parameters were collected for 38 species of shark (62 stocks), comprising five orders and 10 families, as follows: one species in Hemiscylliidae of Orectolobiformes; seven species in Alopiidae, Cetorhinidae, and Lamnidae of Lamniformes; 27 species in Triakidae, Carcharhinidae, and Sphyrnidae of Carcharhiniformes; two species in Squalidae of Squaliformes; and one species in Hexanchidae of Hexanchiformes [44] (S1 and S2 Tables).

As conventional demographic analysis assumes that males are not the limiting factor regulating population growth, this study used data only from females. Where sex-specific parameters were not available, sexes-combined parameters were used. In total, 12 vital parameters were selected. These included five age and growth parameters: asymptotic length (L_∞_), growth coefficient (k), age at zero length (t_0_), maximum age (T_max_), and maximum observed length (L_max_); and seven reproduction parameters: age at maturity (T_m_), reproductive strategy (R), size at maturity (L_m_), size at birth (L_b_), litter size (f), gestation period (G_p_), and reproductive cycle (R_c_). Different studies define vital parameters in slightly different ways. To account for this inconsistency, we used the following definitions:

1. Size at maturity (L_m_): size at 50% maturity, or mean size of mature specimens, or the mean of the maximum and minimum size at maturity if only the range of size at maturity was given.
2. Size at birth (L_b_): the smallest free swimmer, or the mean of the largest full term embryo and the smallest free swimmer.
3. Maximum age (longevity) (T_max_): the maximum ages were assumed as follows: the blacknose shark, *Carcharhinus acronotus* in northern California waters, the northwestern Atlantic and Mexican waters, 26, 20, and 17 yrs respectively; the blue shark in the Northwest Pacific, 18 yrs; basking shark, 49 yrs; and shortfin mako, 41 yrs [19, 45, 46, 47, 48, 49]. The T_max_ of other species was estimated from Taylor’s [50] equation as follow: $T_{max}=t_{0}-\frac{\text{ln}\left(0.05\right)}{k}$
4. Fecundity (f): the mean litter size of pregnant females, or the mean of the maximum and minimum of litter sizes.
5. Maximum observed size (L_max_): the maximum size of observed sharks.
6. Age at maturity (T_m_): the age at 50% maturity, or the mean age of mature specimens, or the mean of the maximum and minimum age at maturity if only the range of age at maturity was given.
7. Reproduction cycle (R_c_): including gestation and resting periods, if only gestation data were available, R_c_ was estimated using data from similar species.

### Input parameters

Large variations in L_b_, L_m_, and L_∞_ were found between different species (S1 and S2 Tables) and this may affect the results of analysis. To eliminate the size-effect in our baseline analysis (scenario 1), we used 7 vital input parameters, namely L_b_, the ratio between size at birth and asymptotic length (L_b_/L_∞_), the ratio between size at maturity and asymptotic length (L_m_/L_∞_), T_max_, T_m_, k, and annual fecundity (f/R_c_). As input parameters may affect the results of multivariate analysis, we simulated two other scenarios for comparison using different input parameters. Five vital parameters, namely L_m_, L_b_, f, k, and T_max_ proposed by Cortés [41] were used in scenario 2, and six parameters (those in scenario 2 pluses one additional parameter, L_∞_) proposed by King and Mcfarlane [38] were used in scenario 3.

### Demographic analysis

The conventional demographic analysis requires an input of natural mortality (M). Thus, Hoenig’s equations [51] were used to estimate the mean M for each stock dependent on the longevity as follows: ln(*M*) = ln(*Z*) = 0.941 − 0.873 * ln(*T*_*max*_), for L_∞_ > 100 cm; ln(*M*) = ln(*Z*) = 1.46 − 1.01 * ln(*T*_*max*_) for L_∞_ < 100 cm [26], where Z is total mortality. Natural mortality approaches Z when the fish stock is unfished or at light exploitation levels. We followed Krebs’s [52] formula to calculate demographic parameters, assuming a sex ratio of 1:1. Since $\sum \frac{1}{2}m_{x}*l_{x}*e^{-rx}=1$, the initial intrinsic rate of population growth, *r*, can be calculated by iteration; net reproductive value per generation ${\text{R}}_{0}=\sum \frac{1}{2}m_{x}*l_{x}*e^{-rx}$, where *m*_*x*_ is fecundity at age *x*, l_*x*_ is the survival rate until age *x*; generation length in years, $\text{G}=\sum \frac{1}{2}m_{x}*l_{x}*e^{-rx}/R_{0}$; the intrinsic rate of natural increase r = ln(R_0_)/*G*; and the finite rate of population increase, λ = e^*r*^. The 95% confidence interval of λ were obtained from 1000 iterations using bootstrap method by randomly selecting M from the following four methods: (1) Hoenig’s equation [51], (2) M = 1.65/*t*_*mat*_ [53], (3) M = 1.6 * k [53], (4) M = −ln(0.01)/*t*_*max*_ [51, 54].

### Multivariate analysis

Due to inconsistencies in measurement units, our PCA used correlation matrices, R, rather than variance-covariance matrices. All parameters were log-transformed and normalized and the eiganvectors and eiganvalues were estimated. A non-parametric multiple dimensional scaling (NMDS) was used to draw the biplot. Vital parameters were reduced to several independent principal components and the scores of principal components were then analyzed using the cluster analysis.

The cluster analysis with Ward's method was used to estimate the scores of the first to third principal components and to draw the tree plot. Species with similar parameter values were grouped together and named according to their shared life history traits. After grouping, the general linear model (GLM) was used to develop an empirical equation for each group describing the relationship between the finite rate of population increase and vital parameters. A GLM was also used to describe the finite rate of population increase for all 62 shark stocks. The Akaike information criterion (AIC) and Bayesian information criterion (BIC) were both used for model selection [55]. A variance inflation factor (VIF) [56] was used to examine the multicollinearity of vital parameters in our multiple regression analysis: $VIF_{j}=\frac{1}{1-R_{xj}^{2}(X_{1},\ldots ,X_{j-1},\;X_{j+1},\ldots ,\;X_{p-1})}$.

Multicollinearity exists among vital parameters when *VIF*_*j*_ ≥ 10, and the parameter can be removed from the regression model.

### Robustness of estimation

We used Jack-knife resampling simulations to estimate the robustness of our empirical equations. For each simulation, we randomly eliminated 1–3 samples from each group and repeated GLM estimations 1000 times. We also estimated the means and standard errors of intercept and coefficient of regression of each of these simulations. To validate the results of our empirical equations, an independent data set including three species which had not been used in developing the equations was substituted into the empirical equations.

## Results

### Vital parameters

#### Age and growth, reproduction, and litter size

For age and growth parameters, the maximum value of L_∞_ was 970 cm TL for the basking shark [19], the minimum was 71.5 cm TL for the spadenose shark, *Scoliodon laticaudus* [18] and the median was 265.4 cm TL. The maximum k value was 0.369 yr^-1^ for the whiskery shark, *Furgaleus macki* [57], the minimum was 0.034 yr^-1^ for the piked dogfish [17] and the median was 0.107 yr^-1^. The minimum L_max_ was 69 cm TL for the spadenose shark [18], the maximum was 970 cm TL for the basking shark [19] (S1 Table) and the median was 242 cm TL.

For reproductive parameters, the maximum L_b_ was 174 cm TL for the pelagic thresher [22], the minimum was 14 cm TL for the spadenose shark [18, 58], and the median was 61 cm TL. Size at maturity, L_m,_ ranged from a minimum of 34 cm TL for the spadenose shark, to a maximum of 500 cm TL for the basking shark [19] with a median of 185 cm TL. The age at maturity ranged from 2 yrs for the spadenose shark to 30 yrs for the sandbar shark [59]. The gestation period ranged from 5 months for the bonnethead shark *Sphyrna tiburo* [60] to 31 months for the basking shark [17], with a median of 12 months.

Thirty of the 62 stocks (48.4%) have a 2-yr reproductive cycle, e.g. the bull shark in northern Mexican waters [61] and the spinner shark [62]; 15 stocks (24.2%) have a 1-yr cycle, e.g. the porbeagle shark, [63], and the Carcharhinid sharks [64]; 17 stocks (27.4%) have a 3-yr cycle, e.g. the school shark, *Galeorhinus galeus* in Brazilian waters [65], and the shortfin mako, *Isurus oxyrinchus* in the northwestern Pacific [16] (S2 Table).

Litter size varies remarkably among species even for the same reproductive trait. For example, for viviparous sharks, the smallest litter size was six for the basking shark [19], while the largest was 82 for the blue shark [20]. A similar situation was found for aplacental viviparous sharks, with litter size ranging from two for the bigeye and pelagic thresher sharks [21, 22] to 55 for the tiger shark, *Galeocerdo cuvier* [66] (S2 Table).

#### The ratios of L_b_/L_∞_, L_m_/L_∞_, and L_b_/L_m_

The L_b_/L_∞_ ratios of the 38 species (62 stocks) ranged from 0.12 to 0.47 with a median value of 0.21. Fifty-two of 62 stocks (83.9%) fell in the range 0.12–0.28 (mean = 0.23), while the remaining 10 stocks (16.1%) were in the range 0.30–0.47. The highest value was 0.47 for the spottail shark *Carcharhinus sorrah* in northern Australia and the blacknose shark *C*. *acronotus* in northwest Atlantic, while the lowest value was for the pike dogfish *Squalus acanthias* in the southeastern Black Sea (S1 Table).

The L_m_/L_∞_ ratios ranged from 0.45 for the thresher shark *Alopias vulpinus* in Californian waters to 0.94 for the whiskery shark *Furgaleus macki* in southwest Australian waters. The median value was 0.68. Thirty-four of 62 stocks (54.8%) fell in the range 0.6–0.8, seventeen (27.4%) had values between 0.45 and 0.59, and eleven stocks (17.7%) were in the range 0.81–0.94.

The L_b_/L_m_ ratios ranged from 0.07 for the sevengill shark, *Notorynchus cepedianus* to 0.86 for the blacknose shark, with a median value of 0.33. Fifty-three of 62 stocks (85.5%) fell in the range 0.2–0.5, two stocks (3.2%) had values between 0.17 and 0.19, and seven stocks (11.3%) were in the range 0.52–0.86 (S1 Table).

#### Maximum age and natural mortality

The maximum age ranged from 7 years for the spottail shark, *Carcharhinus sarrah* to 81 years for the pike dogfish; for 45 of 62 stocks (72.6%) the range was 7–37 years, while 17 (27.4%) fell in the range 38–81 years (S1 Table). Natural mortality rates estimated from Hoenig’s [51] equation range from 0.055 yr^-1^ for the pike dogfish to 0.474 yr^-1^for the spottail shark (S1 Table).

#### Litter size per year

The litter size per year ranged from 1.67 for the longnose spurdog, *Squalus blainville*, in Italian waters to 41 for the blue shark in California, with a median value of 8.5. Fifty of 62 stocks (80.6%) ranged from 1.67–8.06; ten stocks (16.1%) fell in the range 11–19; and two stocks (3.2%) were in the range 39.5–41 (S2 Table).

### Finite rate of population increase

The finite rate of population increase estimated from conventional demographic analysis ranged from 0.929±0.064 for the grey reef shark, *Carcharhinus amblyrhynchos* to 1.470 ± 0.114 for the spadenose shark, *Scoliodon laticaudus*. Thirty-nine of 62 stocks (62.9%) fell in the range 1.0084 ± 0.060–1.1453 ± 0.0722, 19 stocks (30.6%) had values greater than 1.1542 ± 0.0901, and four stocks (6.5%) had values lower than 1 (S3 Table).

### Multivariate analyses

#### Non-parametric multiple dimensional scaling

**Fig 1:**
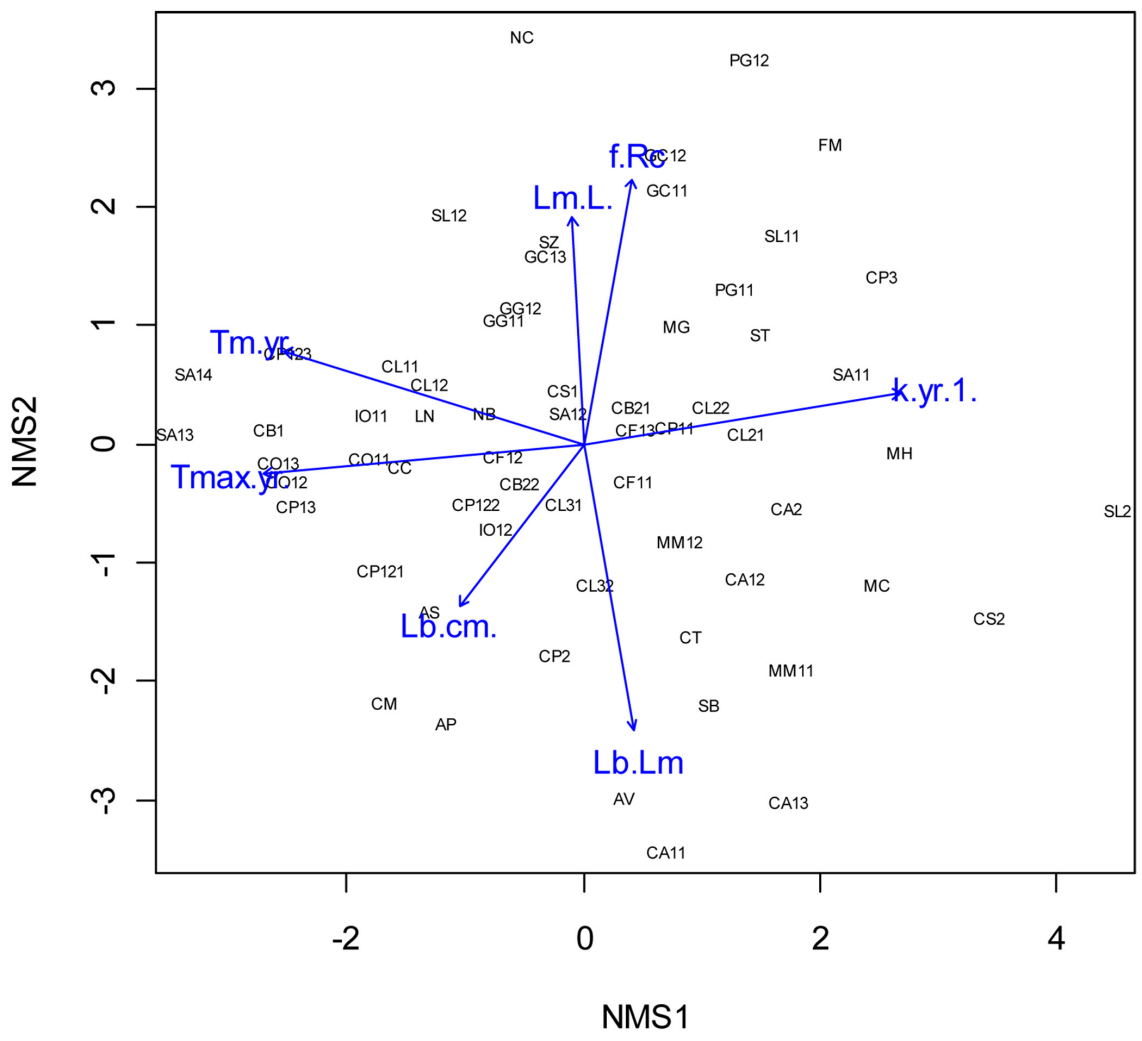
The biplot of two dimensional NMDS. Black labels are species, blue arrows are life history traits.

The bivariate plot of the two dimensional NMDS was showed in Fig 1:

1. In dimension 1, the positive scores represent fast growing species with large k, such as the brown smooth–hound and oceanic whitetip sharks; the negative scores represent late maturing species with large T_max_, and extended longevity, such as the dusky and sandbar sharks.
2. In dimension 2, the positive scores represent species with high L_m_/L_∞_ ratio and high annual fecundity, such as the tiger shark and smooth hammerhead; the negative scores represent species with large size at birth and large L_b_/L_∞_ ratio, such as the three threshers.

#### Cluster analysis and empirical equations of the finite rate of population increase

**Fig 2:**
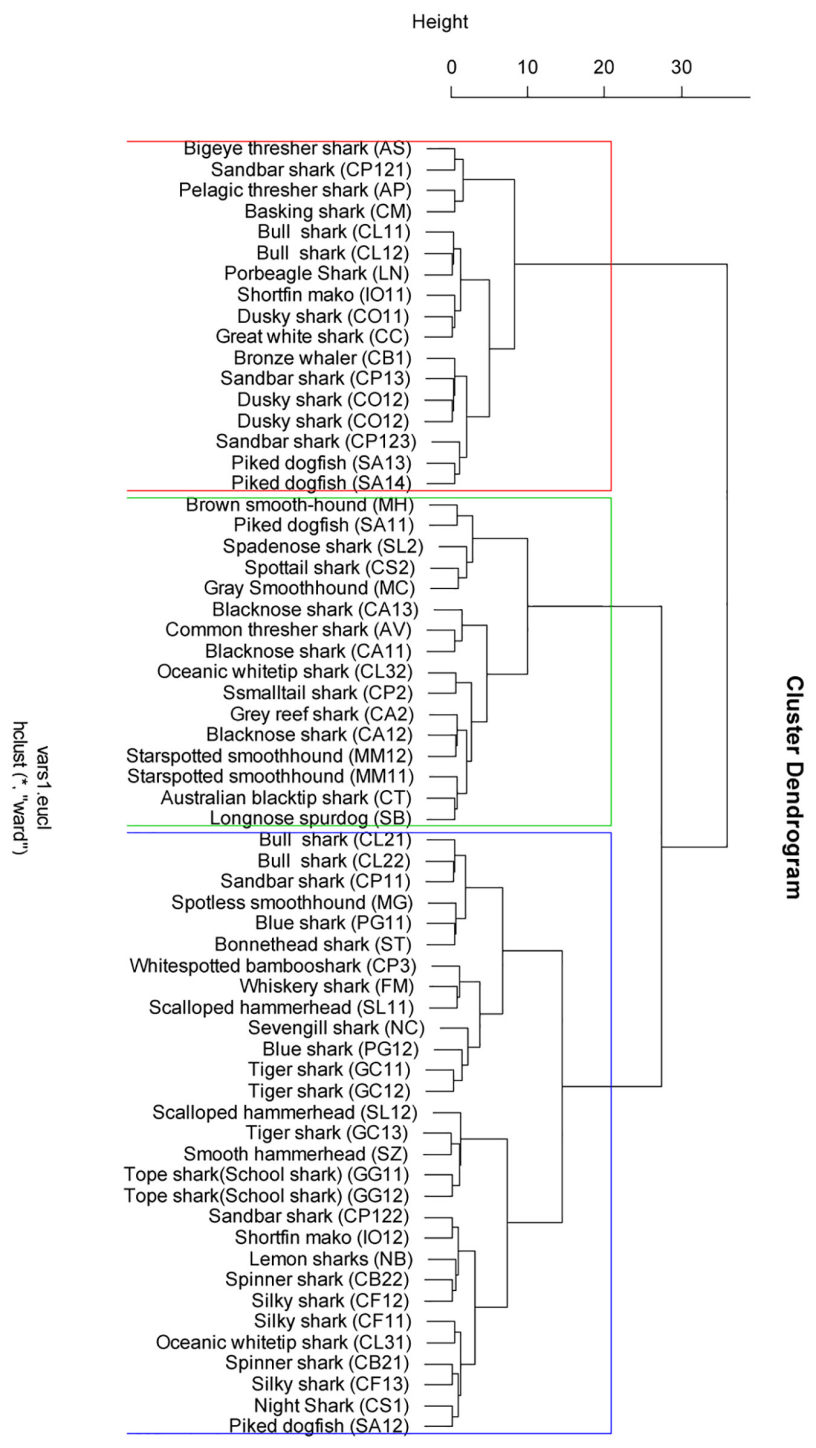
Dendrogram from a cluster analysis of seven vital parameter of 62 stocks from 38 species of sharks. The grouping shows similarities in life history traits among species and stocks from scenario 1.

In scenario 1, three groups were identified based on the cluster analysis (Fig 2):

Group 1: Slow growing species (0.034 yr^-1^ < k < 0.111 yr^-1^) with high maximum age (26 yr < T_max_ < 81 yr). A total of 17 stocks fell into this group, most being large sharks such as the shortfin mako, and dusky shark. The maximum age ranged from 26 yrs for the tiger shark to 81 yrs for the piked dogfish, with the majority of stocks (10) being in the range 28–51 yrs. Longevity, age at maturity and fecundity per year were significant parameters in this group. The empirical equation for estimating the finite rate of population increase is: λ′ = 1.064 + 0.076 * ln(T_*max*_) − 0.128 * ln(T_*m*_) + 0.035 * ln(*f* / *R*_*c*_) (n = 17, r^2^ = 0.97, sd = 0.0070) (Table 1).

**Table 1:** Vital parameters of the species in group 1 from scenario 1.

Obs	Scientific name	Common name	R	L_b_/L_∞_	L_m_/L_∞_	k (yr^-1^)	T_max_ (yr)	T_m_ (yr)	f/R_c_	λ	λ′	|D_i_|^*^
59	*Squalus*. *acanthias* (Canada)	Piked dogfish	ov	0.20	0.72	0.0340	80.81	23.00	1.22	1.0483 ± 0.0625	1.0360	0.01
58	*S*. *acanthias* (NEP)	Piked dogfish	ov	0.17	0.61	0.0360	76.51	29.00	1.18	1.0309 ± 0.0821	1.0050	0.03
8	*Carcharhinus*. *brachyurus* (SAF)	Bronze whaler	v	0.19	0.60	0.0385	74.33	20.00	4.00	1.0919 ± 0.0475	1.0739	0.02
22	*C*. *obscurus* (NWA)	Dusky shark	v	0.22	0.68	0.0390	69.77	21.00	1.83	1.0615 ± 0.0671	1.0512	0.01
27	*C*. *plumbeus* (WAU)	Sandbar shark	v	0.19	0.56	0.0390	71.91	16.20	2.25	1.0862 ± 0.0694	1.0792	0.01
21	*C*. *obscurus* (NAU)	Dusky shark	v	0.22	0.67	0.0430	69.67	19.50	1.42	1.0570 ± 0.0501	1.0555	0.00
26	*C*. *plumbeus* (NWA4)	Sandbar shark	v	0.27	0.81	0.0460	58.67	30.00	2.25	1.0295 ± 0.0591	1.0085	0.02
41	*Isurus oxyrinchus* (NWP)	Shortfin mako	ov	0.18	0.67	0.0498	40.04	20.00	1.85	1.0300 ± 0.0763	1.0565	0.03
20	*C*. *obscurus* (NWP)	Dusky shark	v	0.24	0.68	0.0560	50.08	16.40	2.75	1.0804 ± 0.0743	1.0822	0.00
24	*C*. *plumbeus* (NWA1)	Sandbar shark	v	0.24	0.52	0.0590	45.98	15.50	2.10	1.0706 ± 0.0683	1.0815	0.01
43	*Lamna nasus* (NWA)	Porbeagle shark	ov	0.19	0.70	0.0610	43.21	13.10	2.00	1.0791 ± 0.0711	1.0930	0.01
33	*Cetorhinus maximus*	Basking shark	ov	0.15	0.50	0.0620	48.39	5.00	1.00	1.1441 ± 0.0495	1.1264	0.02
32	*Carcharodon carcharias* (SAF)	Great white shark	ov	0.19	0.63	0.0650	41.69	12.50	2.80	1.1029 ± 0.0630	1.1026	0.00
14	*C*. *leucas* (SAF)	Bull shark	v	0.30	0.84	0.0710	37.07	21.00	2.18	1.0226 ± 0.0723	1.0540	0.03
42	*I*. *oxyrinchus* (California)	Shortfin mako	ov	0.19	0.57	0.0720	37.86	7.50	1.50	1.1213 ± 0.0635	1.1176	0.00
54	*Sphyrna*. *lewini* (NWGM)	Scalloped hammerhead	v	0.15	0.76	0.0730	38.84	15.00	7.50	1.1286 ± 0.0483	1.1279	0.00
40	*Galeorhiuns*. *galeus* (NZ)	Tope shark (School shark)	ov	0.19	0.75	0.0750	36.94	8.00	3.85	1.1905 ± 0.0548	1.1342	0.06
15	*C*. *leucas* (NGM)	Bull shark	v	0.24	0.79	0.0760	36.42	18.00	2.00	1.0351 ± 0.0420	1.0679	0.03
28	*C*. *porosus* (NB)	Smalltail shark	v	0.23	0.51	0.0760	36.14	6.00	1.13	1.1309 ± 0.0810	1.1223	0.01
49	*Negaprion brevirostris* (NEB)	Lemon sharks	v	0.15	0.60	0.0770	36.75	9.70	2.75	1.131 ± 0.04860	1.1165	0.01
10	*C*. *brevipinna* (GM)	Spinner shark	v	0.27	0.68	0.0800	33.61	7.50	2.25	1.1452 ± 0.0907	1.1237	0.02
12	*C*. *falciformis* (NET)	Silky shark	v	0.21	0.65	0.0838	32.99	9.70	2.25	1.1104 ± 0.0855	1.1125	0.00
25	*C*. *plumbeus* (NWA3)	Sandbar shark	v	0.28	0.62	0.0860	30.93	15.50	2.25	1.036 ± 0.05920	1.0828	0.05
39	*G*. *galeus* (NZ)	Tope shark (School shark)	ov	0.18	0.70	0.0860	32.15	14.00	4.03	1.0911 ± 0.0815	1.1049	0.01
18	*C*. *longimanus* (SWEA)	Oceanic whitetip shark	v	0.25	0.65	0.0990	26.87	6.50	1.75	1.1290 ± 0.0640	1.1248	0.00
60	*S*. *blainville* (Italy)	Longnose spurdog	ov	0.13	0.49	0.1020	27.99	5.10	0.83	1.0869 ± 0.0561	1.1245	0.04
38	*G*. *cuvier* (Atlantic)	Tiger shark	ov	0.19	0.72	0.1070	25.65	10.00	13.75	1.1925 ± 0.0861	1.2042	0.01
55	*S*. *zygaena* (NET)	Smooth hammerhead	v	0.15	0.69	0.1108	25.73	11.00	7.50	1.1542 ± 0.0901	1.1484	0.01

R: reproductive strategy, v: viviparity, ov: aplacental viviparity, L_b_/L_∞_: ratio of size at birth and asymptotic length, L_m_/L_∞_: ratio of size at maturity and asymptotic length, k: growth coefficient, T_max_: maximum age, T_m_: age at maturity, f/R_c_: annual fecundity, λ: finite rate of population increase, λ’: λ estimated from empirical equation, *|D_i_| = λ − λ′

Group 2: Fast growing species (0.103 < k < 0.358 yr^-1^), with small T_max_ (9 < T_max_ < 26). A total of 16 stocks fell into this group. The value of k for 11 of 16 stocks (68.75%) fell in the range 0.103–0.18 yr^-1^, with the remaining five stocks (61.25%) ranging from 0.21 to 0.358 yr^-1^. The largest k value was for the spadenose shark (k = 0.358 yr^-1^), while the smallest was for the oceanic whitetip shark (k = 0.103 yr^-1^). T_max_ ranged from 9 yrs for the spadenose shark to 27 yrs for the oceanic whitetip shark. Most species in this group were small size, such as the spotless smoothhound, starspotted smoothhound, and whitespotted bamboo shark. The significant parameters for this group were found to be the ratio between size at maturity and asymptotic length, longevity, age at maturity, growth rate and fecundity per year. The empirical equation for estimating the finite rate of population increase is: λ′=0.984−0.240*ln(LmL∞)+0.142*ln(Tmax)−0.380*ln(Tm)+0.132*ln(f/Rc)(n = 16, r^2^ = 0.95, sd = 0.0359) (Table 2).

**Table 2:** Vital parameters of the species in group 2 from scenario 1.

Obs	Scientific name	Common name	R	L_b_/L_∞_	L_m_/L_∞_	k (yr^-1^)	T_max_ (yr)	T_m_ (yr)	f/R_c_	λ	λ′	|D_i_|^*^
19	*Carcharhinus longimanus* (Pacific)	Oceanic whitetip shark	v	0.21	0.54	0.1030	26.39	4.50	1.50	1.1673 ± 0.0572	1.1266	0.04
46	*Mustelus*. *griseus* (NWT)	Spotless smoothhound	v	0.21	0.58	0.1100	25.02	5.80	7.00	1.3004 ± 0.0585	1.2637	0.04
48	*M*. *manazo* (Tokyo Bay)	Starspotted smoothhound	ov	0.19	0.57	0.1130	23.96	4.50	1.50	1.1604 ± 0.0849	1.1266	0.03
47	*M*. *manazo* (Taiwan)	Starspotted smoothhound	ov	0.26	0.53	0.1240	21.38	2.00	2.55	1.2885 ± 0.054	1.3101	0.02
4	*C*. *acronotus* (NC)	Blacknose shark	v	0.38	0.46	0.1380	25.11	3.50	2.25	1.2545 ± 0.0856	1.2127	0.04
31	*C*. *tilstoni* (NAU)	Australian blacktip shark	v	0.32	0.58	0.1400	18.60	3.50	1.50	1.1764 ± 0.0891	1.1838	0.01
3	*Alopias vulpinus* (California)	Common thresher shark	ov	0.24	0.45	0.1580	17.94	5.00	1.50	1.124 ± 0.0783	1.0979	0.03
51	*Prionace glauca* (NWP)	Blue shark	v	0.14	0.59	0.1614	17.24	4.20	7.25	1.3456 ± 0.107	1.3649	0.02
45	*M*. *californicus* (CC)	Gray smoothhound	v	0.16	0.48	0.1680	16.56	2.10	1.75	1.2562 ± 0.058	1.2736	0.02
56	*Squalus acanthias* (SEBS)	Piked dogfish	ov	0.12	0.61	0.1700	16.89	5.00	1.37	1.101 ± 0.0526	1.0928	0.01
61	*Sphyna tiburo* (NWF)	Bonnethead shark	v	0.21	0.68	0.1800	15.71	4.00	5.50	1.3796 ± 0.0774	1.3090	0.07
6	*C*. *acronotus* (GM)	Blacknose shark	v	0.47	0.55	0.2100	16.50	3.00	2.55	1.3055 ± 0.0871	1.2413	0.06
34	*Chiloscyllitum plagiosum* (NT)	Whitespotted bambooshark	o	0.16	0.70	0.2240	11.50	4.50	4.00	1.1063 ± 0.0478	1.2227	0.12
44	*M*. *henlei* (CC)	Brown smoothhound	v	0.20	0.61	0.2440	10.98	3.00	2.00	1.1453 ± 0.0722	1.2317	0.09
53	*S*. *lewini* (NET)	Scalloped hammerhead	v	0.15	0.72	0.2490	11.62	4.70	6.45	1.2565 ± 0.0602	1.3055	0.05
62	*Scoliodon laticaudus* (India)	Spadenose shark	v	0.20	0.48	0.3580	8.96	1.50	3.75	1.4697 ± 0.1143	1.3849	0.08

R: reproductive strategy, o: ovaprity, v: viviparity, ov: aplacental viviparity, L_b_/L_∞_: ratio of size at birth and asymptotic length, L_m_/L_∞_: ratio of size at maturity and asymptotic length, k: growth coefficient, T_max_: maximum age, T_m_: age at maturity, f/R_c_: annual fecundity, λ: finite rate of population increase, λ’: λ estimated from empirical equation, *|D_i_| = λ − λ′

Group 3: Late-maturing species (L_m_/L_∞_ ≥ 0.67) with moderate T_max_ (T_max_ ≤ 29 yr). L_m_/L_∞_ ranged from 0.67 for the silky shark to 0.94 for the Australian whiskery shark, with 13 of 29 stocks (72.2%) in the range 0.75–0.85. A second characteristic of this group was larger values of f/R_c_ and L_b_/L_∞_. The species with low f/R_c_ have high L_b_/L_∞_ such as the pelagic thresher (f/R_c_ = 1, L_b_/L_∞_ = 0.45) and blacknose shark (f/R_c_ = 1.25, L_b_/L_∞_ = 0.47). Conversely, those with high f/R_c_ have low L_b_/L_∞_ such as the blue shark (f/R_c_ = 20.5, L_b_/L_∞_ = 0.16), sevengill shark (f/R_c_ = 19.75, L_b_/L_∞_ = 0.15), and tiger shark (f/R_c_ = 13.75, L_b_/L_∞_ = 0.15). The empirical equation for estimating the finite rate of population increase is: λ′ = 1.377 − 0.057 * ln(L_*b*_) + 0.169 * ln(L_*b*_/L_∞_) − 0.261 * ln(L_*m*_/L_∞_) + 0.160 * ln(*T*_*max*_) − 0.340 * ln(*T*_*m*_) + 0.152 * ln(*f*/*R*_*c*_) (n = 29, r^2^ = 0.93, sd = 0.0297) (Table 3). Since VIF < 10, this indicates an absence of multicollinearity for the three equations.

**Table 3:** Vital parameters of the species in group 3 from scenario 1.

Obs	Scientific name	Common name	R	L_b_/L_∞_	L_m_/L_∞_	k (yr^-1^)	T_max_ (yr)	T_m_ (yr)	f/R_c_	λ	λ′	|D_i_|^*^
11	*Carcharhinus falciformis* (Pacific)	Silky shark	v	0.25	0.67	0.1480	18.48	6.50	2.13	1.0977 ± 0.0857	1.1176	0.02
7	*C*. *amblyrhynchos*	Grey reef shark	v	0.32	0.73	0.2940	12.00	7.00	1.25	0.9285 ± 0.0641	0.9444	0.02
30	*C*. *sorrah* (NAU)	Spottail shark	v	0.47	0.75	0.3400	6.91	2.50	1.50	0.9666 ± 0.0489	0.9838	0.02
1	*Alopias pelagicus* (NEP)	Pelagic thresher shark	ov	0.45	0.75	0.0850	27.57	8.60	1.00	1.0493 ± 0.042	1.0886	0.04
29	*C*. *signatus* (NEB)	Night shark	v	0.25	0.76	0.1140	23.58	10.00	3.13	1.0974 ± 0.0854	1.0755	0.02
9	*C*. *brevipinna* (NET)	Spinner shark	v	0.23	0.77	0.1510	17.85	7.80	2.13	1.0619 ± 0.0741	1.0614	0.00
13	*C*. *falciformis* (NWGM)	Silky shark	v	0.25	0.77	0.1530	17.38	8.00	1.75	1.0385 ± 0.0446	1.0341	0.00
57	*Squalus acanthias* (NWA)	Piked dogfish	ov	0.27	0.80	0.1057	25.44	12.10	1.10	0.9973 ± 0.0432	0.9862	0.01
2	*Alopias superciliosus* (NET)	Bigeye thresher shark	ov	0.35	0.80	0.0920	28.35	12.85	1.00	1.0084 ± 0.06	0.9687	0.04
16	*C*. *limbatus* (SAF)	Blacktip shark	v	0.23	0.81	0.2100	13.17	7.00	1.00	0.9397 ± 0.0604	1.0000	0.06
37	*Galeocerdo cuvier* (GM)	Tiger shark	ov	0.15	0.82	0.1840	15.15	8.00	13.75	1.1787 ± 0.0757	1.1953	0.02
17	*C*. *limbatus* (TB)	Blacktip shark	v	0.27	0.82	0.1970	14.05	6.50	2.00	1.0315 ± 0.0618	1.0333	0.00
23	*C*. *plumbeus* (NET)	Sandbar shark	v	0.30	0.82	0.1700	15.32	7.85	1.89	1.0165 ± 0.0424	0.9852	0.03
52	*Prionace glauca* (NEP)	Blue shark	v	0.16	0.83	0.2230	12.63	6.50	20.50	1.2958 ± 0.0774	1.3001	0.00
50	*Notorynchus cepedianus* (NEP)	Sevengill shark	ov	0.15	0.84	0.1070	28.00	15.95	19.75	1.1382 ± 0.0707	1.1722	0.03
5	*C*. *acronotus* (NWA)	Blacknose shark	v	0.47	0.89	0.1800	19.25	4.50	1.25	1.1119 ± 0.0865	1.1102	0.00
36	*G*. *cuvier* (Hawaii)	Tiger shark	ov	0.19	0.90	0.1550	18.71	5.00	11.50	1.3624 ± 0.0723	1.3370	0.03
35	*Furgaleus macki* (SWA)	Whiskery shark	ov	0.27	0.94	0.3690	11.50	6.50	9.50	1.1603 ± 0.0595	1.0870	0.07

R: reproductive strategy, v: viviparity, ov: aplacental viviparity, L_b_/L_∞_: ratio of size at birth and asymptotic length, L_m_/L_∞_: ratio of size at maturity and asymptotic length, k: growth coefficient, T_max_: maximum age, T_m_: age at maturity, f/R_c_: annual fecundity, λ: finite rate of population increase, λ’: λ estimated from empirical equation, *|D_i_| = λ − λ′

To sum up, Group 1 species reach asymptotic length at an older age (T_max_ > 25 yr); than Group 2 species (T_max_ < 13 yr). Only Groups 1 and 2 have overlapping values of L_m_/L_∞_ and T_max_ between the groups.

The empirical equation for 62 stocks combined is λ′ = 1.116 − 0.029 * ln(L_*b*_) + 0.108 * ln(*L*_*b*_/*L*_*m*_) − 0.141 * ln(L_*m*_/L_∞_) + 0.154 * ln(*T*_*max*_) − 0.242 * ln(*T*_*m*_) + 0.119 * ln(*f*/*R*_*c*_) (n = 62, r^2^ = 0.77, sd = 0.0654).

The results of Jack-knife simulations indicated the robustness of the empirical equations for Groups 1–3, as well as the combined equation (Table 4). Using 1000 simulations, the coefficients of variation for each parameter mean of Groups 1–3 and the combined-group equation were 4.28%–21.48%, 3.17%–13.55%, 4.11%–97.27%, and 3.45%–12.28%, respectively. Moreover, the 95% confidence intervals of the parameter means also indicate that the parameters of each of the four equations are statistically significant and robust (Table 4).

**Table 4:** The partial regression coefficients and their coefficient of variation of the empirical equations for Groups 1 to 3 and combined-group.

Group	Intercept	L_b_	L_b_/L_∞_	L_m_/L_∞_	T_max_	T_m_	f/R_c_
Group1	1.06 (4.11%)	——	——	——	0.08 (10.13%)	-0.13 (14.09%)	0.04 (97.27%)
Group2	0.98 (7.01%)	——	-0.24 (12.34%)	——	0.14 (5.90%)	-0.38 (4.28%)	0.13 (21.48%)
Group3	1.38 (3.17%)	-0.06 (13.55%)	0.17 (10.79%)	-0.26 (13.19%)	0.26 (13.19%)	-0.34 (8.78%)	0.15 (3.54%)
Combined	1.12 (3.45%)	-0.03 (9.87%)	0.11 (11.28%)	-0.14 (11.18%)	0.15 (12.28%)	-0.24 (9.12%)	0.12 (6.77%)

In scenario 2, two groups were identified by cluster analysis. No significant relationship was found between vital parameters and λ´ for cluster 1 (p = 0.123; n = 13), while only fecundity was correlated to λ´ for cluster 2: *λ*' = 1.1136 + 0.0038*f*, (*p* = 0.037; *n* = 49). Two groups were also identified for cluster 3 but no significant relationship was found between vital parameters and λ´ for either cluster in this scenario.

#### Validation of empirical equations

**Fig 3:**
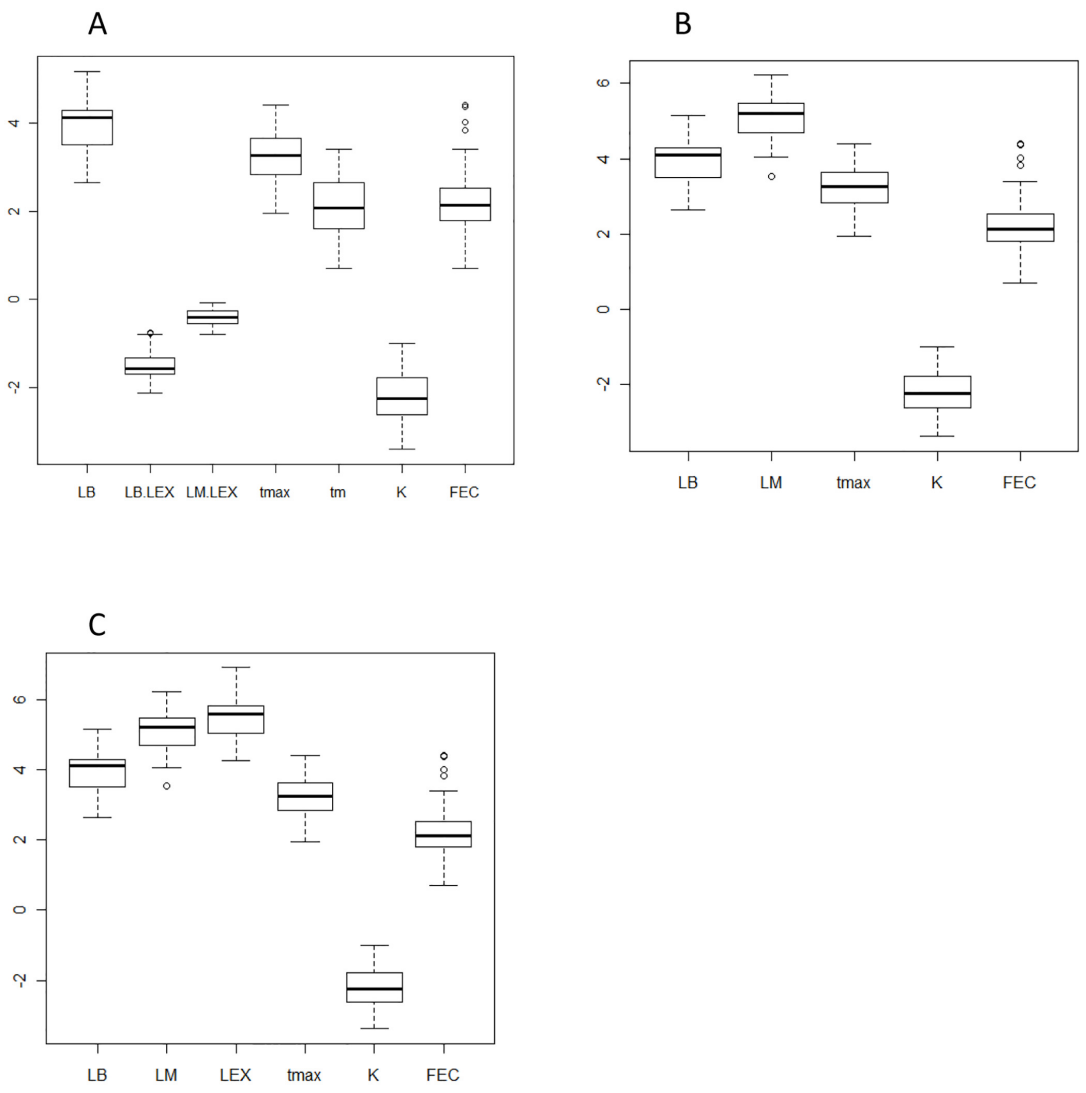
Box plot of vital parameters for Groups 1, 2, and 3. (Figs 3A, 3B, and 3C, respectively). L_b_: size at birth, L_m_: size at maturity, L_inf_: asymptotic length, L_b_/ L_inf_: ratio of L_b_ and L_inf_, T_max_: maximum age, K: growth coefficient, f/R_C_: annual fecundity.

The independent data set for validation the results of our empirical equations included the vital parameters for Groups 1–3 species, leopard shark *Triakis semifasciata*, grey nurse shark *Carcharias taurus* and gummy shark *Mustelus antarcticus*. The predicted values of λ’ for each group (1.154, 1.002, and 1.268) showed good agreement with those derived from conventional demographic analysis (1.199, 0.977, and 1.239) (Fig 3, Tables 1–3). High correlations between predicted λ’ and λ for Groups 1–3 and combined equation (r^2^ = 0.97, 0.95, 0.93 and 0.77, respectively) were found.

## Discussion

In this study, we used the vital parameters of 62 shark stocks to develop empirical equations to estimate population increase rates. Although these data have been filtered by existed knowledge, neither quantitative analysis nor rigorous criterion was used in choosing these data set. Therefore, the inconsistence of data quality may occurred in this study. Thorson et al. [67] mentioned that meta-analyses employing hierarchical models could account for experimental design differences, covariates and non-random assignment of study sites to treatment and control groups, and would likely increase precision for effect-size estimates. Hierarchical models should be included in the analysis in the future.

### Factors affect estimate ofλ

Several factors may affect the estimate of λ.

#### Body length

To deal with inconsistencies in length measurement found in the literature, this study converted all lengths to TL, other than those which were already designated as total length. This standardization improved the quality of our results.

#### Growth coefficient

Inconsistencies were also found in the literature with respect to age determination, even for the same species. For example, Branstetter [68] reported annual band pair formation for the scalopped hammerhead in the northwestern Mexican waters, while Chen et al. [69], Anislado-Tolentino et al. [70] and Kotas et al. [71] reported a biennial formation for the same species in the northeastern Taiwanese waters, southern coast of Mexico, and Brazil waters, respectively. Similarly, for shortfin mako, Pratt and Casey [72] reported biannual band pair deposition while Cailliet et al. [73] suggested an annual deposition. Neer et al. [74] have indicated that the accuracy of age determination significantly affects stock assessment. To ensure as wide a range of data as possible, and allow for the above-mentioned inconsistencies, this study collected and analyzed growth parameters for the same species in different waters.

Under Branstetter's [75] categorization, k values of 0.05–0.10 yr^-1^ indicate slow growth, 0.10–0.20 yr^-1^ indicate moderate growth, and 0.20–0.50 yr^-1^ indicate rapid growth. In our study, examples of slow growth species included the dusky shark (k = 0.043 yr^-1^; [76]), shortfin mako (k = 0.05 yr^-1^, [49]), and porbeagle shark (k = 0.061 yr^-1^; [77]); examples of moderate growth species included the blacknose shark (k = 0.114 yr^-1^; [78]), spinner shark (k = 0.151 yr^-1^; [62]), and blue shark (k = 0.1614 yr^-1^; [48]); and examples of rapid growth species included the whitespotted bamboo shark (k = 0.224 yr^-1^; [79]), and grey reef shark (k = 0.294 yr^-1^; [41]). As the growth parameters used in this study covered a wide range of growth rates, our results derived from this study can be applied to the species with different growth rates.

#### Reproduction cycle

Wourms [80] identified three basic types of reproductive cycle: (1) reproduction occurring throughout the year; (2) a partially defined annual cycle with one or two peaks during the year; and (3) a well-defined annual or biennial cycle. The pelagic thresher shark [22] is an example of a first-category type, while the epaulette shark, *Hemiscyllium ocellatum* [81], falls into the second category. Examples of a third-category type include the shortfin mako, with a 3-year reproduction cycle (2 years of gestation and 1 year of resting) [16], and the spinner shark, with a 2-year reproduction cycle (1 year of gestation and 1 year of resting) [62]. The result of shark stock assessment based on demographic analysis is affected by both the gestation and resting periods [49]. The estimates in this study take both gestation and resting periods into account and therefore, we believe, provide more accurate and realistic results.

#### Litter size

The litter size may be underestimated when it was estimated based on the carcasses at the fish market. Embryos may be lost during the capture process for viviparous or aplacental viviparous sharks which result in the underestimation of litter size. Branstetter [82] and Bonfil [83] documented that female silky sharks may have aborted pups from uterus during capture if litter sizes less than 5 pups. To reduce the uncertainty, future study should focus on collecting more reliable litter size information from on board observation.

#### The ratio of L_b_/L_∞_

Branstetter [75] documented a trade-off between litter size and size at birth. Species with small litter size compensate by having a larger L_b_ and higher L_b_/L_∞_. Joung [84] stated that the ratio of L_b_/L_∞_ ranged from 0.15–0.35 for most elasmobranches. With few exceptions, the species in this study were in this range. Usually, a negative relation between f/R_c_ and L_b_/L_∞_ was evident. For example, the blue shark (L_b_/L_∞_ = 0.14;f/R_c_ = 14.5); blacknose shark (L_b_/L_∞_ = 0.38;f/R_c_ = 4.5), pelagic thresher shark (L_b_/L_∞_ = 0.45;f/R_c_ = 2) and spottail shark (L_b_/L_∞_ = 0.47; f/R_c_ = 3). Unlike the studies by Cortés [41] and King and Mcfarlane [38], L_b_/L_∞_ instead of L_b_/L_max_ was used as an input parameter in this study. Given the high correlation between L_max_ and L_∞_ for the 62 stocks in this study (r = 0.937), our approach should be considered an acceptable alternative.

#### The ratio of L_m_/L_∞_

Since there is considerable variation in size at maturity among species (34–336.6 cm TL, S2 Table), analysis based on the input parameter L_m_ might produce bias. This study therefore used the ratio of L_m_/L_∞_ instead. Compagno [58] stated that for sharks, L_m_/L_∞_ was 0.6–0.8. Pratt and Casey [85] also concluded that the L_m_/L_∞_ of most elasmobranches is above 0.5. The 38 species analyzed in this study included all the maturing types defined by Joung [84]. That is, early maturing species (L_m_/L_∞_ < 0.6), such as the thresher shark (L_m_/L_∞_ = 0.45), and blacknose shark (L_m_/L_∞_ = 0.46) moderate maturing species (0.6 < L_m_/L_∞_ < 0.8), such as the dusky shark (L_m_/L_∞_ = 0.68), whitespotted bamboo shark (L_m_/L_∞_ = 0.7), and silky shark (L_m_/L_∞_ = 0.77); and late maturing species (L_m_/L_∞_ > 0.8), such as the whiskery shark (L_m_/L_∞_ = 0.94), tiger shark (L_m_/L_∞_ = 0.9) and sevengill shark (L_m_/L_∞_ = 0.84).

#### Maximum age

Skomal and Natanson [86] pointed out that using the maximum observed age to represent the maximum age might result in underestimates. Equations developed by Taylor [50], Fabens [87] and Pauly [88] are commonly used to estimate maximum age. In this study, the values estimated from the latter two equations were much higher than the maximum observed age, and therefore Taylor’s [50] equation was used. Froese and Binohlan [89] suggested that for most sharks the total length was in the range 100–300 cm with a ratio L_max_/L_∞_ of 0.97–0.987. Chen and Yuan [37] claimed that the maximum age estimated from Taylor’s [50] equation is more reasonable than those derived from other equations. In this study, apart from the blacknose, basking, blue, and shortfin mako, for which maximum ages were adopted from the literature, the T_max_ was estimated using Taylor [50], and we believe the results to be reasonable.

### Estimate of natural mortality

The natural mortality of marine animals is difficult to estimate. Ohsumi et al. [90] proposed a linear relationship between *L*_∞_ and longevity to estimate the natural mortality of the minke whale. Pauly’s [91] empirical equation between *M* and *L*_∞_, *k*, and habitat mean water temperature has been widely used to estimate *M* for teleost fishes. Several attempts have been made by other authors e.g., Peterson and Wroblewski [92], Chen and Watanabe [93] and Jensen [53], but these studies have also focused on teleosts. As little is known of the life history parameters of sharks, Hoenig’s [51] relationship between longevity and total mortality has been adopted by many authors [26, 27, 28, 29, 30, 32, 94]. Hoenig [51] put forward three empirical equations, of which Cortés [26] suggested using the equation for marine mammals to represent sharks larger than 100 cm and the equation for teleosts to represent sharks smaller than 100 cm. Our study follows this suggestion. Chen and Yuan [37], on the other hand, used Hoenig’s [51] equation for teleosts to estimate M for sharks greater than 100 cm. We believe this might have led to an overestimation. Recently, Then et al. [95] suggested that a new t_max_-based estimator is better than other empirical equations in natural mortality estimation. Although this method has not been tried in this study, since the empirical equations [51] we used is also t_max_-based equation and is the most frequently used method for elasmobranchs, we believe our estimates are robust.

### Estimation of λ

Cortés [34] and Chen and Yuan [37] have applied demographic analysis to sharks using vital parameter data. The estimates of λ in this study using conventional demographic methods were comparable to those of Cortés [34]. The λ value of sharks derived by Chen and Yuan [37] may be an underestimate as they calculated natural mortality using Pauly’s [91] method, which is not suitable (an overestimation) for sharks.

Cortés [33] estimated intrinsic population growth rate through stochastic demographic analysis by applying Monte Carlo simulations based on T_m_, T_max_, fecundity, and M. In this study, both gestation and resting periods were included in the calculation of *λ* and stochastic effects have also been considered in estimating the confidence interval of *λ*. Therefore, we believe this produces a reasonable estimate.

#### Input parameters

The reproductive cycle was not used as an input parameter in scenarios 2 and 3, but was included in scenario 1. In addition, variations in size among species were reduced by using the ratios L_b_/L_∞_ and L_m_/L_∞_ rather than L_b_ and L_m_. We believe the output of scenario 1 is more reasonable than those derived from scenarios 2 and 3.

### Cluster analysis

The groups defined in scenario 1 have distinct life history characteristics. With a few exceptions, the 62 stocks can be correctly categorized based on their life history parameters. In contrast, distinct life history characteristics did not appear in scenarios 2 and 3. Therefore, we believe that the results obtained in scenario 1 are likely to be more realistic than those in scenarios 2 and 3.

### Validation and application of empirical equation

The high correlation between predicted λ’ and λ for Groups 1–3 and combined equation and the randomly distributed residuals, suggest that the empirical equations developed in this study can predict λ precisely than other models, and also need fewer vital parameters in Groups 1 and 2. It therefore provides an effective and efficient approach to shark management. The predicted values of λ’ for each group of the independent data set showed good agreement with those derived from conventional demographic analysis suggesting that the empirical equations can be applied to predict λ for other shark species. In other words, the empirical equations derived in this study reduce the uncertainties, and increase the accuracy, of population increase estimates, even without the inclusion of a natural mortality variable.

Bayesian production model has been used in shark stock assessment [96, 97, 98, 99]. One of the key input prior for this model is the intrinsic population growth rate r (r = ln(λ)). Our empirical equations, which can accurately estimate the λ can enhance the ability of stock assessment.

### Uncertainty of vital parameter

The reproduction cycle is one of the most ambiguous vital parameters. This information is available for only 21 of 62 stocks in the literature. For the remaining stocks estimates were made using data on gestation periods and by referring to the reproduction cycle of similar species. However, discrepancies may exist due to variations in geography and these may result in inaccurate estimates of annual litter size. Most age at maturity and maximum age values have been estimated from the VBGE, but many uncertainties have been found, including sample size, specimen size range, band reading etc. These uncertainties may lead to inaccurate estimates of λ in empirical equations.

### Management measures

Based on life history characteristics, conventional studies have categorized fish strategies into r and K types. Fish with r strategy are small size, early-maturing, and have a short life span. Those with K strategy are large size, late-maturing, and have an extended life span. These strategies correspond to the management measures of teleost and chondrichthyan fishes. Walker [100] suggested that a K rather than r strategy should be adopted for shark management and marine mammals. Also, management measures should vary according to the catch and stock status of different species and areas.

### Recommendations for management

In this study, management recommendation was given only for scenario 1, as this was considered more realistic than the other two scenarios. Group 1 stocks are mostly large, slow-growing species with small litter size. Given that these populations recover slowly even when they experience slight overfishing, a protection of adults or TAC management measure has been suggested e.g., school shark, *Galeorhinus galeus* [101], and shortfin mako [49]. Group 2 stocks are mostly small, fast-growing species with large litter size. Regular stock assessment with management of the fishing area and fishing season closure has been suggested [39]. Group 3 stocks are mostly late-maturing species which recover slowly. A reduce of catch or TAC management has been suggested e.g., thresher shark, *Alopias vulpinus* [4] and pelagic thresher [102, 103].

## Conclusions

Conventional stock assessment analysis requires fishing effort or other biological information. In this study, we provide a new approach to the accurate estimation of the finite rate of population increase. The empirical equations developed herein not only provide accurate predictions of λ but also reduce estimate bias resulting from parameter uncertainties. We believe that this is an effective and efficient approach to the implementation of precautionary shark management measures. However, we recognize that these equations could be improved further. Our study considered only 38 of 498 shark species existing worldwide, [15]. Therefore, our estimates may not take into account all the various life history traits of different shark species. Moreover, potentially influential environmental factors such as water temperature, water depth, and salinity were not considered in this study. To improve the accuracy and usefulness of these empirical equations, we suggest that future studies be directed toward these areas.

## Supporting Information

S1 Table Age and growth parameters for the 62 stocks (38 species) of sharks used in this study. (DOCX)

S2 Table Reproductive parameters for the 62 stocks (38 species) of sharks used in this study. (DOCX)

S3 Table Finite population increase rate derived from demographic analysis for 62 stocks (38 species) of sharks. (DOCX)
