# Supplementary material for: Estimating Finite Rate of Population Increase for Sharks Based on Vital Parameters
Source: PLoS One. 2015 Nov 17;10(11):e0143008. doi: 10.1371/journal.pone.0143008 (PMC4648575; doi:10.1371/journal.pone.0143008)
Supplement: S1 Table — (DOCX) [file pone.0143008.s001.docx]

S1-1 Table Age and growth parameters for the 62 stocks (38 species) of sharks used in this study.

| **Obs** | **Scientific name** | **Common name** | **L_max_ (cm)** | **L_∞_ (cm)** | **k (yr^-1^)** | **t_0_** | **T_max_ (yr)** | **Source** |
| --- | --- | --- | --- | --- | --- | --- | --- | --- |
| 1 | *Alopias pelagicus* (NEP) | Pelagic thresher shark | 365.18 | 382.94 | 0.0850 | -7.67 | 27.57 | [1] |
| 2 | *A. superciliosus* (NET) | Bigeye thresher shark | 422.00 | 422.00 | 0.0920 | -4.21 | 28.35 | [2] |
| 3 | *A. vulpinus* (California) | Common thresher shark | 609.60 | 636.00 | 0.1580 | -1.02 | 17.94 | [3] |
| 4 | *Carcharhinus acronotus* (NC) | Blacknose shark | 205.64 | 241.28 | 0.1380 | -2.68 | 25.11 | [4] |
| 5 | *C. acronotus* (NWA) | Blacknose shark | 154.00 | 180.16 | 0.1800 | -4.07 | 19.25 | [5] |
| 6 | *C. acronotus* (GM) | Blacknose shark | 175.95 | 200.32 | 0.2100 | -1.58 | 16.50 | [6] |
| 7 | *C. amblyrhynchos* | Grey reef shark | 187.00 | 187.00 | 0.2940 | − | 12.00 | [7] |
| 8 | *C. brachyurus* (SAF) | Bronze whaler | 288.00 | 384.80 | 0.0385 | -3.48 | 74.33 | [8] |
| 9 | *C. brevipinna* (NET) | Spinner shark | 274.00 | 288.20 | 0.1510 | -1.99 | 17.85 | [9] |
| 10 | *C. brevipinna* (GM) | Spinner shark | 224.90 | 271.19 | 0.0800 | -3.84 | 33.61 | [10] |
| 11 | *C. falciformis* (Pacific) | Silky shark | 279.57 | 287.73 | 0.1480 | -1.76 | 18.48 | [11] |
| 12 | *C. falciformis* (NET) | Silky shark | 256.00 | 332.00 | 0.0838 | -2.76 | 32.99 | [12] |
| 13 | *C. falciformis* (NWGM) | Silky shark | 267.00 | 290.50 | 0.1530 | -2.20 | 17.38 | [13] |
| 14 | *C. leucas* (SAF) | Bull shark | 221.00 | 230.00 | 0.0710 | -5.12 | 37.07 | [14] |
| 15 | *C. leucas* (NGM) | Bull shark | 268.00 | 285.00 | 0.0760 | -3.00 | 36.42 | [15] |
| 16 | *C. limbatus* (SAF) | Blacktip shark | 245.72 | 262.27 | 0.2100 | -1.10 | 13.17 | [16] |
| 17 | *C. limbatus* (TB) | Blacktip shark | 180.00 | 195.00 | 0.1970 | -1.15 | 14.05 | [17] |
| 18 | *C. longimanus* (SWEA) | Oceanic whitetip shark | 250.00 | 284.90 | 0.0990 | -3.39 | 26.87 | [18] |
| 19 | *C. longimanus* (Pacific) | Oceanic whitetip shark | 263.80 | 341.68 | 0.1030 | -2.70 | 26.39 | [19] |
| 20 | *C. obscurus* (NWP) | Dusky shark | 364.00 | 415.70 | 0.0560 | -3.42 | 50.08 | [20] |
| 21 | *C. obscurus* (NAU) | Dusky shark | 324.58 | 418.65 | 0.0430 | − | 69.67 | [21] |
| 22 | *C. obscurus* (NWA) | Dusky shark | 333.21 | 420.61 | 0.0390 | -7.04 | 69.77 | [22] |
| 23 | *C. plumbeus* (NET) | Sandbar shark | 210.00 | 210.00 | 0.1700 | -2.30 | 15.32 | [23] |
| 24 | *C. plumbeus* (NWA1) | Sandbar shark | 234.00 | 263.30 | 0.0590 | -4.80 | 45.98 | [24] (1980-1981 sample) |
| 25 | *C. plumbeus* (NWA3) | Sandbar shark | 174.00 | 220.50 | 0.0860 | -3.90 | 30.93 | [24] (1980-1981 sample) |
| 26 | *C. plumbeus* (NWA4) | Sandbar shark | 201.60 | 223.38 | 0.0460 | -6.45 | 58.67 | [25] |

L_max_: maximum observed length, L_∞_: asymptotic length, k: growth coefficient, t_o_: theoretical age at zero length, T_max_: maximum age, −: none.

S1-2 Table Age and growth parameters for the 62 stocks (38 species) of sharks used in this study.

| **Obs** | **Scientific name** | **Common name** | **L_max_ (cm)** | **L_∞_ (cm)** | **k (yr^-1^)** | **t_0_** | **T_max_ (yr)** | **Source** |
| --- | --- | --- | --- | --- | --- | --- | --- | --- |
| 27 | *C. plumbeus* (WAU) | Sandbar shark | 223.97 | 279.39 | 0.0390 | -4.90 | 71.91 | [26] |
| 28 | *C. porosus* (NB) | Ssmalltail shark | 101.00 | 136.30 | 0.0760 | -3.28 | 36.14 | [27] |
| 29 | *C. signatus* (NEB) | Night shark | 242.00 | 265.40 | 0.1140 | -2.70 | 23.58 | [28] |
| 30 | *C. sorrah* (NAU) | Spottail shark | 115.00 | 123.90 | 0.3400 | -1.90 | 6.91 | [29] |
| 31 | *C. tilstoni* (NAU) | Australian blacktip shark | 168.90 | 194.20 | 0.1400 | -2.80 | 18.60 | [29] |
| 32 | *Carcharodon carcharias* (SAF) | Great white shark | 600.00 | 685.75 | 0.0650 | -4.40 | 41.69 | [30] |
| 33 | *Cetorhinus maximus* | Basking shark | 970.00 | 1000 | 0.0620 | − | 48.39 | [31] |
| 34 | *Chiloscyllitum plagiosum* (NT) | Whitespotted bambooshark | 85.00 | 93.20 | 0.2240 | − | 11.50 | [32] |
| 35 | *Furgaleus macki* (SWA) | Whiskery shark | 137.85 | 134.58 | 0.3690 | -0.54 | 11.50 | [15] |
| 36 | *Galeocerdo cuvier* (Hawaii) | Tiger shark | 350.00 | 335.00 | 0.1550 | -0.62 | 18.71 | [33] |
| 37 | *G. cuvier* (GM) | Tiger shark | 355.00 | 388.00 | 0.1840 | -1.13 | 15.15 | [34] |
| 38 | *G. cuvier* (Atlantic) | Tiger shark | 381.00 | 440.00 | 0.1070 | -2.35 | 25.65 | [34] |
| 39 | *Galerorhinus galeus* (NZ) | Tope shark (School shark) | 168.00 | 179.20 | 0.0860 | -2.68 | 32.15 | [35] |
| 40 | *G. galeus* (NZ) | Tope shark (School shark) | 155.00 | 163.00 | 0.0750 | -3.00 | 36.94 | [36] |
| 41 | *Isurus oxyrinchus* (NWP) | Shortfin mako | 375.00 | 413.80 | 0.0498 | − | 40.04 | [37, 38] |
| 42 | *I. oxyrinchus* (California) | Shortfin mako | 321.00 | 321.00 | 0.0720 | -3.75 | 37.86 | [3] |
| 43 | *Lamna nasus* (NWA) | Porbeagle shark | 328.82 | 348.94 | 0.0610 | -5.90 | 43.21 | [39] |
| 44 | *Mustelus henlei* (CC) | Brown smoothhound | 100.00 | 97.70 | 0.2440 | -1.30 | 10.98 | [40] |
| 45 | *M. californicus* (CC) | Gray Smoothhound | 124.00 | 154.40 | 0.1680 | -1.27 | 16.56 | [40] |
| 46 | *M. griseus* (NWT) | Spotless smoothhound | 89.00 | 124.71 | 0.1100 | -2.21 | 25.02 | [41] |
| 47 | *M. manazo* (Taiwan) | Starspotted smoothhound | 86.50 | 113.70 | 0.1240 | -2.78 | 21.38 | [42] |
| 48 | *M. manazo* (Tokyo Bay) | Starspotted smoothhound | 107.00 | 134.10 | 0.1130 | -2.55 | 23.96 | [43, 44] |
| 49 | *Negaprion brevirostris* (NEB) | Lemon sharks | 340.00 | 399.90 | 0.0770 | -2.16 | 36.75 | [45] |
| 50 | *Notorynchus cepedianus* (NEP) | Sevengill shark | 253.00 | 275.00 | 0.1070 | − | 28.00 | [46] |
| 51 | *Prionace glauca* (NWP) | Blue shark | 323.00 | 322.70 | 0.1614 | -1.33 | 17.24 | [47] |
| 52 | *P. glauca* (NEP) | Blue shark | 245.00 | 265.00 | 0.2230 | -0.80 | 12.63 | [3] |

L_max_: maximum observed length, L_∞_: asymptotic length, k: growth coefficient, t_o_: theoretical age at zero length, T_max_: maximum age, −: none.

S1-3 Table Age and growth parameters for the 62 stocks (38 species) of sharks used in this study.

| **Obs** | **Scientific name** | **Common name** | **L_max_ (cm)** | **L_∞_ (cm)** | **k (yr^-1^)** | **t_0_** | **T_max_ (yr)** | **Source** |
| --- | --- | --- | --- | --- | --- | --- | --- | --- |
| 53 | *Sphyrna lewini* (NET) | Scalloped hammerhead | 324.00 | 319.72 | 0.2490 | -0.41 | 11.62 | [48] |
| 54 | *S. lewini* (NWGM) | Scalloped hammerhead | 309.00 | 329.00 | 0.0730 | -2.20 | 38.84 | [13] |
| 55 | *S. zygaena* (NET) | Smooth hammerhead | 324.00 | 375.20 | 0.1108 | -1.31 | 25.73 | [49] |
| 56 | *Squalus acanthias* (SEBS) | Piked dogfish | 136.00 | 145.00 | 0.1700 | -0.73 | 16.89 | [50] |
| 57 | *S. acanthias* (NWA) | Piked dogfish | 90.00 | 100.50 | 0.1057 | -2.90 | 25.44 | [51] |
| 58 | *S. acanthias* (NEP) | Piked dogfish | 130.00 | 152.90 | 0.0360 | -6.70 | 76.51 | [52] |
| 59 | *S. acanthias* (Canada) | Piked dogfish | 93.00 | 129.00 | 0.0340 | -7.30 | 80.81 | [52] |
| 60 | *S. blainville* (Italy) | Longnose spurdog | 92.00 | 117.90 | 0.1020 | -1.38 | 27.99 | [53] |
| 61 | *Sphyna tiburo* (NWF) | Bonnethead shark | 110.00 | 139.80 | 0.1800 | -0.93 | 15.71 | [54] |
| 62 | *Scoliodon laticaudus* (India) | Spadenose shark | 69.00 | 71.50 | 0.3580 | 0.59 | 8.96 | [25, 55] |

L_max_: maximum observed length, L_∞_: asymptotic length, k: growth coefficient, t_o_: theoretical age at zero length, T_max_: maximum age.
