# Supplementary material for: Estimating Finite Rate of Population Increase for Sharks Based on Vital Parameters
Source: PLoS One. 2015 Nov 17;10(11):e0143008. doi: 10.1371/journal.pone.0143008 (PMC4648575; doi:10.1371/journal.pone.0143008)
Supplement: S2 Table — (DOCX) [file pone.0143008.s002.docx]

S2-1 Table Reproductive parameters for the 62 stocks (38 species) of sharks used in this study.

| **Obs** | **Scientific name** | **Common name** | **R** | **L_b_ (cm)** | **L_m_ (cm)** | **T_m_ (yr)** | **f** | **G_p_ (month)** | **R_c_ (yr)** | **L_b_/L_∞_** | **L_m_/L_∞_** | **L_b_/L_m_** | **f/R_c_** | **Source** |
| --- | --- | --- | --- | --- | --- | --- | --- | --- | --- | --- | --- | --- | --- | --- |
| 1 | *Alopias pelagicus* (NEP) | Pelagic thresher shark | ov | 174.00 | 287.00 | 8.60 | 2.0 | 12.0 | 1 | 0.45 | 0.75 | 0.61 | 2.00 | [1] |
| 2 | *A. superciliosus* (NET) | Bigeye thresher shark | ov | 148.70 | 336.58 | 12.85 | 2.0 | 12.0 | 1 | 0.35 | 0.80 | 0.44 | 2.00 | [56] |
| 3 | *A. vulpinus* (California) | Common hresher shark | ov | 150.00 | 287.50 | 5.00 | 3.0 | 9.0 | 1 | 0.24 | 0.45 | 0.52 | 3.00 | [3] |
| 4 | *Carcharhinus acronotus* (NC) | Blacknose shark | v | 92.50 | 109.95 | 3.50 | 4.5 | 9.0 | 1 | 0.38 | 0.46 | 0.84 | 4.50 | [4] |
| 5 | *C. acronotus* (NWA) | Blacknose shark | v | 85.08 | 159.79 | 4.50 | 5.0 | 11.0 | 2 | 0.47 | 0.89 | 0.53 | 2.50 | [5] |
| 6 | *C. acronotus* (GM) | Blacknose shark | v | 94.58 | 110.00 | 3.00 | 4.5 | 9.50 | 1 | 0.47 | 0.55 | 0.86 | 4.5 | [6, 57] |
| 7 | *C. amblyrhynchos* | Grey reef shark | v | 60.00 | 137.00 | 7.00 | 5.0 | 12.0 | 2 | 0.32 | 0.73 | 0.44 | 2.50 | [7] |
| 8 | *C. brachyurus* (SAF) | Bronze whaler | v | 74.00 | 229.00 | 20.00 | 16.0 | 12.0 | 2 | 0.19 | 0.60 | 0.32 | 8.00 | [8] |
| 9 | *C. brevipinna* (NET) | Spinner shark | v | 67.50 | 222.50 | 7.80 | 8.5 | 11.0 | 2 | 0.23 | 0.77 | 0.30 | 4.25 | [9] |
| 10 | *C. brevipinna* (GM) | Spinner shark | v | 72.02 | 185.00 | 7.50 | 9.0 | 13.5 | 2 | 0.27 | 0.68 | 0.39 | 4.50 | [25] |
| 11 | *C. falciformis* (Pacific) | Silky shark | v | 73.36 | 193.48 | 6.50 | 8.5 | 12.0 | 2 | 0.25 | 0.67 | 0.38 | 4.25 | [11] |
| 12 | *C. falciformis* (NET) | Silky shark | v | 69.50 | 215.00 | 9.70 | 9.0 | 12.0 | 2 | 0.21 | 0.65 | 0.32 | 4.50 | [12] |
| 13 | *C. falciformis* (NWGM) | Silky shark | v | 72.00 | 225.00 | 8.00 | 7.0 | 12.0 | 2 | 0.25 | 0.77 | 0.32 | 3.50 | [13] |
| 14 | *C. leucas* (SAF) | Bull shark | v | 68.50 | 193.00 | 21.00 | 8.7 | 11.0 | 2 | 0.30 | 0.84 | 0.35 | 4.35 | [14] |
| 15 | *C. leucas* (NGM) | Bull shark | v | 67.50 | 225.00 | 18.00 | 8.0 | 11.5 | 2 | 0.24 | 0.79 | 0.30 | 4.00 | [15] |
| 16 | *C. limbatus* (SAF) | Blacktip shark | v | 60.30 | 212.37 | 7.00 | 6.0 | 14.0 | 3 | 0.23 | 0.81 | 0.28 | 2.00 | [16] |
| 17 | *C. limbatus* (TB) | Blacktip shark | v | 52.29 | 160.00 | 6.50 | 8.0 | 12.0 | 2 | 0.27 | 0.82 | 0.33 | 4.00 | [17] |
| 18 | *C. longimanus* (SWEA) | Oceanic whitetip shark | v | 70.00 | 185.00 | 6.50 | 7.0 | 12.0 | 2 | 0.25 | 0.65 | 0.38 | 3.50 | [18] |
| 19 | *C. longimanus* (Pacific) | Oceanic whitetip shark | v | 69.85 | 181.61 | 4.50 | 6.0 | 10.5 | 2 | 0.21 | 0.54 | 0.38 | 3.00 | [19] |
| 20 | *C. obscurus* (NWP) | Dusky shark | v | 101.00 | 281.00 | 16.40 | 11.0 | 13.0 | 2 | 0.24 | 0.68 | 0.36 | 5.50 | [20] |
| 21 | *C. obscurus* (NAU) | Dusky shark | v | 92.10 | 278.95 | 19.50 | 8.5 | 16.0 | 3 | 0.22 | 0.67 | 0.33 | 2.83 | [21] |
| 22 | *C. obscurus* (NWA) | Dusky shark | v | 92.50 | 284.12 | 21.00 | 11.0 | 16.0 | 3 | 0.22 | 0.68 | 0.33 | 3.67 | [22] |
| 23 | *C. plumbeus* (NET) | Sandbar shark | v | 62.50 | 172.50 | 7.85 | 7.5 | 11.0 | 2 | 0.30 | 0.82 | 0.36 | 3.77 | [58] |
| 24 | *C. plumbeus* (NWA1) | Sandbar shark | v | 63.00 | 136.00 | 15.50 | 8.4 | 10.5 | 2 | 0.24 | 0.52 | 0.46 | 4.20 | [24] (1980-1981 sample) |
| 25 | *C. plumbeus* (NWA3) | Sandbar shark | v | 61.00 | 136.00 | 15.50 | 9.0 | 10.5 | 2 | 0.28 | 0.62 | 0.45 | 4.50 | [24] (1980-1981 sample) |
| 26 | *C. plumbeus* (NWA4) | Sandbar shark | v | 60.00 | 179.82 | 30.00 | 9.0 | 12.0 | 2 | 0.27 | 0.81 | 0.33 | 4.50 | [25] |

R: reproductive strategy, v: viviparity, ov: aplacental viviparity, T_max_: maximum age, f: litter size, G_p_: gestation period, R_c_: reproduction cycle, L_b_/L_∞_: ratio of size at birth and asymptotic length, L_m_/L_∞_: ratio of size at maturity and asymptotic length, L_b_/L_m_: ratio of size at birth and length at maturity, f/R_c_: annual fecundity.

S2-2 Table Reproductive parameters for the 62 stocks (38 species) of sharks used in this study.

| **Obs** | **Scientific name** | **Common name** | **R** | **L_b_ (cm)** | **L_m_ (cm)** | **T_m_ (yr)** | **f** | **G_p_ (month)** | **R_c_ (yr)** | **L_b_/L_∞_** | **L_m_/L_∞_** | **L_b_/L_m_** | **f/R_c_** | **Source** |
| --- | --- | --- | --- | --- | --- | --- | --- | --- | --- | --- | --- | --- | --- | --- |
| 27 | *C. plumbeus* (WAU) | Sandbar shark | v | 53.12 | 157.08 | 16.20 | 9.0 | 10.0 | 2 | 0.19 | 0.56 | 0.34 | 4.50 | [26] |
| 28 | *C. porosus* (NB) | Ssmalltail shark | v | 31.00 | 70.00 | 6.00 | 4.5 | 10.0 | 2 | 0.23 | 0.51 | 0.44 | 2.25 | [27] |
| 29 | *C. signatus* (NEB) | Night shark | v | 66.80 | 202.50 | 10.00 | 12.5 | 12.0 | 2 | 0.25 | 0.76 | 0.33 | 6.25 | [28] |
| 30 | *C. sorrah* (NAU) | Spottail shark | v | 58.00 | 92.50 | 2.50 | 3.0 | 10.0 | 1 | 0.47 | 0.75 | 0.63 | 3.00 | [59] |
| 31 | *C. tilstoni* (NAU) | Australian blacktip shark | v | 63.00 | 112.50 | 3.50 | 3.0 | 10.0 | 1 | 0.32 | 0.58 | 0.56 | 3.00 | [59] |
| 32 | *Carcharodon carcharias* (SAF) | Great white shark | ov | 130.31 | 429.30 | 12.50 | 14.0 | 18.0 | 2.5 | 0.19 | 0.63 | 0.30 | 5.60 | [60] |
| 33 | *Cetorhinus maximus* | Basking shark | ov | 150.00 | 500.00 | 5.00 | 6.0 | 31.2 | 3 | 0.15 | 0.50 | 0.30 | 2.00 | [31] |
| 34 | *Chiloscyllitum plagiosum* (NT) | Whitespotted bambooshark | o | 15.02 | 64.90 | 4.50 | 8.0 | - | 1 | 0.16 | 0.70 | 0.23 | 8.00 | [61] |
| 35 | *Furgaleus macki* (SWA) | Whiskery shark | ov | 36.77 | 125.96 | 6.50 | 19.0 | 8.0 | 1 | 0.27 | 0.94 | 0.29 | 19.00 | [62] |
| 36 | *Galeocerdo cuvier* (Hawaii) | Tiger shark | ov | 63.50 | 300.00 | 5.00 | 46.0 | 14.5 | 3 | 0.19 | 0.90 | 0.21 | 15.33 | [33] |
| 37 | *G. cuvier* (GM) | Tiger shark | ov | 60.00 | 317.50 | 8.00 | 55.0 | 14.5 | 3 | 0.15 | 0.82 | 0.19 | 18.33 | [34] |
| 38 | *G. cuvier* (Atlantic) | Tiger shark | ov | 85.00 | 317.50 | 10.00 | 55.0 | 14.5 | 3 | 0.19 | 0.72 | 0.27 | 18.33 | [34] |
| 39 | *Galerorhinus galeus* (NZ) | Tope shark (School shark) | ov | 33.00 | 125.00 | 14.00 | 24.2 | 12.0 | 3 | 0.18 | 0.70 | 0.26 | 8.06 | [35] |
| 40 | *G. galeus* (NZ) | Tope shark (School shark) | ov | 30.30 | 123.00 | 8.00 | 23.1 | 12.0 | 3 | 0.19 | 0.75 | 0.25 | 7.70 | [63] |
| 41 | *Isurus oxyrinchus* (NWP) | Shortfin mako | ov | 74.00 | 278.00 | 20.00 | 11.1 | 24.0 | 3 | 0.18 | 0.67 | 0.27 | 3.70 | [37, 38] |
| 42 | *I. oxyrinchus* (California) | Shortfin mako | ov | 60.50 | 182.80 | 7.50 | 9.0 | 16.5 | 3 | 0.19 | 0.57 | 0.33 | 3.00 | [3] |
| 43 | *Lamna nasus* (NWA) | Porbegle shark | ov | 65.50 | 245.21 | 13.10 | 4.0 | 8.5 | 1 | 0.19 | 0.70 | 0.27 | 4.00 | [39] |
| 44 | *Mustelus henlei* (CC) | Brown smooth-hound | v | 20.00 | 60.00 | 3.00 | 4.0 | 6.0 | 1 | 0.20 | 0.61 | 0.33 | 4.00 | [40] |
| 45 | *M. californicus* (CC) | Gray Smoothhound | v | 25.00 | 74.00 | 2.10 | 3.5 | 10.5 | 1 | 0.16 | 0.48 | 0.34 | 3.50 | [40] |
| 46 | *M. griseus* (NWT) | Spotless smoothhound | v | 26.00 | 72.95 | 5.80 | 14.0 | 9.5 | 1 | 0.21 | 0.58 | 0.36 | 14.00 | [41] |
| 47 | *M. manazo* (Taiwan) | Starspotted smoothhound | ov | 30.00 | 60.00 | 2.00 | 5.1 | 10.0 | 2 | 0.26 | 0.53 | 0.50 | 2.55 | [42] |
| 48 | *M. manazo* (Tokyo Bay) | Starspotted smoothhound | ov | 25.00 | 76.00 | 4.50 | 6.0 | 11.5 | 2 | 0.19 | 0.57 | 0.33 | 3.00 | [43, 44] |
| 49 | *Negaprion brevirostris* (NEB) | Lemon sharks | v | 61.00 | 240.00 | 9.70 | 11.0 | 11.0 | 2 | 0.15 | 0.60 | 0.25 | 5.50 | [45] |
| 50 | *Notorynchus cepedianus* (NEP) | Sevengill shark | ov | 40.00 | 231.00 | 15.95 | 79.0 | 12.0 | 2 | 0.15 | 0.84 | 0.17 | 39.50 | [64] |
| 51 | *Prionace glauca* (NWP) | Blue shark | v | 45.00 | 189.00 | 4.20 | 29.0 | 10.0 | 2 | 0.14 | 0.59 | 0.24 | 14.50 | [65] |
| 52 | *P. glauca* (NEP) | Blue shark | v | 43.50 | 220.00 | 6.50 | 82.0 | 10.5 | 2 | 0.16 | 0.83 | 0.20 | 41.00 | [3] |

R: reproductive strategy, o: ovaprity, v: viviparity, ov: aplacental viviparity, T_max_: maximum age, f: litter size, G_p_: gestation period, R_c_: reproduction cycle, L_b_/L_∞_: ratio of size at birth and asymptotic length, L_m_/L_∞_: ratio of size at maturity and asymptotic length, L_b_/L_m_: ratio of size at birth and length at maturity, f/R_c_: annual fecundity.

S2-3 Table Reproductive parameters for the 62 stocks (38 species) of sharks used in this study.

| **Obs** | **Scientific name** | **Common name** | **R** | **L_b_ (cm)** | **L_m_ (cm)** | **T_m_ (yr)** | **f** | **G_p_ (month)** | **R_c_ (yr)** | **L_b_/L_∞_** | **L_m_/L_∞_** | **L_b_/L_m_** | **f/R_c_** | **Source** |
| --- | --- | --- | --- | --- | --- | --- | --- | --- | --- | --- | --- | --- | --- | --- |
| 53 | *Sphyrna lewini* (NET) | Scalloped hammerhead | v | 48.50 | 230.00 | 4.70 | 25.8 | 10.0 | 2 | 0.15 | 0.72 | 0.21 | 12.90 | [66] |
| 54 | *S. lewini* (NWGM) | Scalloped hammerhead | v | 49.00 | 250.00 | 15.00 | 30.0 | 12.0 | 2 | 0.15 | 0.76 | 0.20 | 15.00 | [13] |
| 55 | *S. zygaena* (NET) | Smooth hammerhead | v | 55.00 | 259.40 | 11.00 | 30.0 | 10.0 | 2 | 0.15 | 0.69 | 0.21 | 15.00 | [67] |
| 56 | *Squalus acanthias* (SEBS) | Piked dogfish | ov | 17.50 | 88.00 | 5.00 | 8.2 | 21.0 | 3 | 0.12 | 0.61 | 0.20 | 2.73 | [50] |
| 57 | *S. acanthias* (NWA) | Piked dogfish | ov | 27.00 | 79.90 | 12.10 | 6.6 | 21.0 | 3 | 0.27 | 0.80 | 0.34 | 2.20 | [68] |
| 58 | *S. acanthias* (NEP) | Piked dogfish | ov | 26.00 | 93.50 | 29.00 | 7.1 | 21.0 | 3 | 0.17 | 0.61 | 0.28 | 2.37 | [52] |
| 59 | *S. acanthias* (Canada) | Piked dogfish | ov | 26.20 | 93.50 | 23.00 | 7.3 | 21.0 | 3 | 0.20 | 0.72 | 0.28 | 2.43 | [52] |
| 60 | *S. blainville* (Italy) | Longnose spurdog | ov | 15.50 | 57.50 | 5.10 | 5.0 | 17.0 | 3 | 0.13 | 0.49 | 0.27 | 1.67 | [53] |
| 61 | *Sphyna tiburo* (NWF) | Bonnethead shark | v | 29.70 | 94.40 | 4.00 | 11.0 | 5.0 | 1 | 0.21 | 0.68 | 0.31 | 11.00 | [54] |
| 62 | *Scoliodon laticaudus* (India) | Spadenose shark | v | 14.00 | 34.00 | 1.50 | 7.5 | 5.5 | 1 | 0.20 | 0.48 | 0.41 | 7.50 | [25, 55] |

R: reproductive strategy, v: viviparity, ov: aplacental viviparity, T_max_: maximum age, f: litter size, G_p_: gestation period, R_c_: reproduction cycle, L_b_/L_∞_: ratio of size at birth and asymptotic length, L_m_/L_∞_: ratio of size at maturity and asymptotic length, L_b_/L_m_: ratio of size at birth and length at maturity, f/R_c_: annual fecundity.

**References**

1. Liu KM, Chen CT, Liao LH, Joung SJ (1999) Age, growth, and reproduction of the pelagic thresher shark, *Alopias pelagicus*, in the northwestern Pacific. Copeia 1999: 68-74.
2. Liu KM, Chen CT, Chiang PJ (1998). Age and growth estimates of the bigeye thresher shark, *Alopias superciliosus*, in northeastern Taiwan waters. Fish Bull 96 (3): 482-491.
3. Cailliet GM, Bedford DW (1983) The biology of three pelagic sharks from California waters, and their emerging fisheries: a review. Reports of Calif Coop Ocean Fish Invest 24: 57-69.
4. Schwartz FJ (1984) Occurrence, abundance, and biology of the blacknose shark, *Carcharhinus acronotus*, in North Carolina. Northeast Gulf Sci 7: 29-47.
5. Driggers WB, Carlson JK, Cullum B, Dean JM, Oakley D, Ulrich G (2004) Age and growth of the blacknose shark, *Carcharhinus acronotus*, in the western North Atlantic Ocean with comments on regional variation in growth rates. Environ Biol Fishes 71: 171-178.
6. Carlson JK, Cortés E, Johnson AG (1999) Age and growth of the blacknose shark, *Carcharhinus acronotus*, in the Eastern Gulf of Mexico. Copeia 1999: 684-691.
7. Cope JM (2006) Exploring intraspecific life history patterns in sharks. Fish Bull 104: 311-320.
8. Walter JP, Ebert DA (1991) Preliminary estimates of age of the bronze whaler, *Charhinus brachyurus*, (Chondrichthyes: Carcharhinidae) from southern Africa, with a review of some life history parameters. South Afr J Mar Sci 10: 37-44.
9. Joung SJ, Liao YY, Liu KM, Chen CT, Leu LC (2005) Age, growth, and reproduction of the spinner shark, *Carcharhinus brevipinna*, in the northeastern waters of Taiwan. Zool Stud 44(1): 102-110.
10. Carlson JK, Braemore IE (2005) Growth dynamics of the spinner shark (*Carcharhinus brevipinna*) off the United States southeast and Gulf of Mexico coasts: a comparison of methods. Fish Bull 103: 280-291.
11. Oshitani S, Nakano H, Tanaka S (2003) Age and growth of silky shark *Carcharhinus falciformis* from the Pacific Ocean. Fish Sci 69: 456-464.
12. Joung SJ, Chen CT, Lee HH, Liu KM (2008) Age, growth, and reproduction of silky sharks, *Carcharhinus falciformis*, in northeastern Taiwan waters. Fish Res 90(1-3): 78-85. doi:10.1016/j.fishres.2007.09.025
13. Branstetter S (1987) Age, growth and reproductive biology of the silky shark, *Carcharhinus falciformis*, and the scalloped hammerhead, *Sphyrna lewini*, from the northwestern Gulf of Mexico. Environ Biol Fishes 19(3): 161-173.
14. Wintner SP, Dudley SFJ, Kistnasamy N, Everett B (2002) Age and growth estimates for the Zamberzi shark, *Carcharhinus leucas*, from the east coast of South Africa. Mar Freshwater Res 53: 557-566.
15. Branstetter S, Stiles R (1987) Age and growth estimates of the bull shark, *Carcharhinus leucas*, from the northern Gulf of Mexico. Environ Biol Fishes 20(3): 169-181.
16. Wintner SP, Cliff G (1996) Age and growth determination of the blacktip shark, *Carcharhinus limbatus*, from the east coast of South Africa. Fish Bull 94: 135-144.
17. Killam KA, Parsons GR (1989) Age and growth of the blacktip shark, *Carcharhinus limbatus*, near Tampa Bay, Florida. Fish Bull 87: 845-857.
18. Lessa R, Santana FM, Paglerani R (1999) Age, growth and stock structure of the oceanic whitetip shark, *Carcharhinus longimanus*, from the southwestern equatorial Atlantic. Fish Res 42: 21-30.
19. Seki T, Taniuchi T, Nakano H, Shimizu M (1998) Age, growth and reproduction of the oceanic whitetip shark from the Pacific Ocean. Fish Sci 64 (1): 14-20.
20. Chen JH (2004) Fishery biology of the dusky shark, *Carcharhinus obscurus* in Northwest Pacific. M. Sc. Thesis, National Taiwan Ocean University, Keelung, Taiwan
21. Simpfendorfer CA, McAuley RB, Chidlow J, Unsworth P (2002) Validated age and growth of the dusky shark, *Carcharhinus obscurus*, from Western Australian waters. Mar Freshwater Res 53: 567-573. doi:10.1071/mf01131
22. Natanson LJ, Casey JG, Kohler NE (1995) Age and growth estimates for the dusky shark, *Carcharhinus obscurus*, in the western North Atlantic Ocean. Fish Bull 93: 116-126.
23. Joung SJ, Liao YY, Chen CT (2004) Age and growth of sandbar shark, *Carcharhinus plumbeus*, in northeastern Taiwan waters. Fish Res 70(1): 83-96.
24. Sminkey TR, Musick JA (1995) Age and growth of the sandbar shark, *Carcharhinus plumbeus*, before and after population depletion. Copeia 1995: 871-883.
25. Casey JG, Natanson LJ (1992) Revised estimates of age and growth of the sandbar shark (*Carcharhinus plumbeus*) from the western North Atlantic. Can J Fish Aquat Sci 49: 1474-1477.
26. McAuley RB, Simpfendorfer CA, Hyndes GA, Allison RR, Chidlow JA, Newman SJ, et al. (2006) Validated age and growth of the sandbar shark, *Carcharhinus plumbeus* (Nardo 1827) in the waters off western Australia. Environ Biol Fishes 77: 385-400. doi:10.1007/s10641-006-9126-0.
27. Lessa R, Santana FM (1998) Age determination and growth of the smalltail shark, *Carcharhinus porosus*, from northern Brazil. Mar Freshwater Res 49: 705-711.
28. Santana FM, Lessa R (2004) Age determination and growth of the night shark (*Carcharhinus signatus*) off the northeastern Brazilian coast. Fish Bull 102:156-167.
29. Davenport S, Stevens JD (1988) Age and growth of two commercially important sharks (*Carcharhinus tilsoni* and *C. sorrah*) from Northern Australia. Aust J Mar Freshwater Res 39: 417-433.
30. Wintner SP, Cliff G (1999) Age and growth determination of the white shark, *Carcharodon carcharias*, from the east coast of South Africa. Fish Bull 97: 153-169.
31. Pauly D (2002) Growth and mortality of the basking shark *Cetorhinus maximus* and their implications for management of whale sharks *Rhincodon typus*. Paper presented at the Elasmobranch Biodiversity, Conservation and Management. Proceedings of the International Seminar and Workshop, Sabah, Malaysia, July 1997.
32. Chen WK, Chen PC, Liu KM, Wang SB (2007) Age and growth estimates of the whitespotted bamboo shark, *Chiloscyllium plagiosum*, in the northern waters of Taiwan. Zool Stud 46(1): 92-102.
33. Randall JE (1992) Review of the biology of the tiger shark (*Galeocerdo cuvier*). Aust J Mar Freshwater Res 43: 21-31.
34. Branstetter S, Musick JA, Colvocoresses JA (1987) A comparison of the age and growth of the tiger shark, *Galeocerdo cuvier*, from off Virginia and from the northwestern gulf of Mexico. Fish Bull 85(2): 269-279.
35. Francis MP, Mulligan KP (1998) Age and growth of New Zealand school shark, *Galeorhinus galeus*. NZ J Mar Freshwater Res 32: 427-440.
36. Ferreira BP, Vooren CM (1991). Age, growth, and structure of vertebra in the school shark *Galeorhinus galeus* (Linnaeus, 1758) from southern Brazil. Fish Bull 89: 19-31.
37. Joung SJ, Hsu HH (2005) Reproduction and embryonic development of the shortfin mako, *Isurus* *oxyrinchus* Rafinesque, 1810, in the northwestern Pacific Zool Stud 44(4): 487-496.
38. Chang JH, Liu KM (2009) Stock assessment of the shortfin mako shark, *Isurus oxyrinchus*, in the Northwest Pacific Ocean using per-recruit and virtual population analyses. Fish Res 98: 92-103. doi:10.1016/j.fishres.2009.04.005
39. Jensen CF, Natanson LJ, Pratt HL, Kohler NE, Campana SE (2002). The reproductive biology of the porbeagle shark (*Lamna nasus*) in the western North Atlantic Ocean. Fish Bull 100: 727-738.
40. Yudin KG, Cailliet GM (1990) Age and growth of the gray smoothhound, *Mustelus californicus*, and the brown smoothhound, *M. henlei*, sharks from central California. Copeia 1990: 191-204.
41. Wang TM, Chen CT (1982) Reproduction of smooth dogfish, *Mustelus griseus*, in northwestern Taiwan waters. J Fish Soc Taiwan (8): 23-26.
42. Yamaguchi A, Taniuchi T, Shimizu M (1998) Geographic variation in growth of the starspotted dogfish, *Mustelus manazo,* from five localities in Japan and Taiwan. Fish Sci 64: 732-739.
43. Yamaguchi A, Taniuchi T, Shimizu M (1996) Age and growth of the starspotted dogfish *Mustelus manazo* from Tokyo Bay, Japan. Fish Sci 62 (6): 919-922.
44. Yamaguchi A, Taniuchi T, Shimizu M (1997) Reproductive biology of the starspotted dogfish *Mustelus manazo* from Tokyo Bay, Japan. Fish Sci 63 (6): 918-922.
45. Freitas RHA, Rosa RS, Gruber SH, Wetherbee BM (2006) Early growth and juvenile population structure of lemon sharks *Negaprion brevirostris* in the Atol das Rocas Biological Reserve, off north-east Brazil. J Fish Biol 68: 1319-1332.
46. Dykhuizen GV, Mollet HF (1992) Growth, age estimation and feeding of captive sevengill sharks, *Notorynchus cepedianus*, at the Monterey Bay Aquarium. Aust J Mar Freshwater Res 43: 297-318.
47. Huang JC (2006) Age and growth of the blue shark, *Prionace glauca* in the Northwest Pacific. M Sc. Thesis, National Taiwan Ocean University, Keelung, Taiwan.
48. Chen CT, Leu TC, Joung SJ, Lo NCH (1990) Age and growth of the scalloped hammerhead *Sphyrna lewini* in northeastern Taiwan waters. Pac Sci 44(2): 156-170.
49. Chou YG (2004) Age and growth of the smooth hammerhead, *Sphyrna zygaena* in the northeastern Taiwan waters. M. Sc. Thesis, National Taiwan Ocean University, Keelung, Taiwan
50. Avsar D (2001) Age, growth, reproduction and feeding of the spurdog (*Squalus acanthias* Linnaeus, 1758) in the South-eastern Black Sea. Estuar Coast Shelf Sci 52: 269-278. doi:10.1006/ecss.2000.0749
51. Beamish R, McFarlane GA (1985) Annulus development of the second dorsal spine of the spiny dogfish (*Squalus acanthias*) and its validity for age determination. Can J Fish Aquat Sci 42: 1799-1805.
52. Ketchen KS (1975) Age and growth of dogfish *Squalus acanthias* in British Columbia waters. J Fish Res Board Can 32(1): 43-59.
53. Cannizzaro L, Rizzo P, Levi D, Gancitano S (1995) Age determination and growth of *Squalus blainvillei* (Risso, 1826). Fish Res 23: 113-125.
54. Lombardi-Carlson LA, Cortés E, Parsons GR, Charles A (2003).Latitudinal variation in life-history traits of bonnethead sharks, *Sphyrna tiburo,* (Carcharhiniformes : Sphyrnidae) from the eastern Gulf of Mexico. Mar Freshwater Res 54(7): 875 – 883.
55. Devadoss P (1998) Growth and population parameters of the spade nose shark, *Scoliodon laticaudus* from Calicut coast. Indian J Fish 45(1): 29-34.
56. Chen CT, Liu KM, Chang YC (1997) Reproductive biology of the bigeye thresher shark, *Alopias superciliosus,* (Lowe, 1839)(Chondrichthyes: Alopiidae), in the northwestern Pacific. Ichthyol Res 44 (3): 227-235.
57. Sulilowski JA, Driggers WB, Ford TS, Boonstra RK, Carlson JK (2007) Reproductive cycle of the blacknose shark *Carcharhinus acronotus* in the Gulf of Mexico. J Fish Biol 70 (2): 428-440.
58. Joung SJ, Chen CT (1995). **Reproduction in the sandbar shark, *Carcharhinus plumbeus*, in the waters off northeastern Taiwan.** Copeia (3): 659-665.
59. Stevens JD, Wiley PD (1986) Biology of two commercially important Carcharhinid sharks from Northern Australia. Aust J Mar Freshwater Res 37: 671-688.
60. Gilmore RG (1993) Reproductive biology of lamnoid sharks. Environ Biol Fishes 38: 95-114.
61. Chen WK, Liu KM (2006) Reproductive biology of whitespotted bamboo shark *Chiloscyllium plagiosum* in northern waters off Taiwan. Fish Sci 72: 1215-1224.
62. Simpfendorfer CA, Unsworth P (1998) Reproductive biology of the whiskery shark, *Furgaleus macki*, off south-western Australia. Mar Freshwater Res 49: 687-693.
63. Peres MB, Vooren CM (1991) Sexual development, reproductive cycle, and fecundity of the school shark *Galeorhinus galeus* off Southern Brazil. Fish Bull 89: 655-667.
64. Lucifora LO, Menni RC, Escalante AH (2005) Reproduction, abundance and feeding habits of the broadnose sevengill shark *Notorynchus cepedianus* in north Patagonia, Argentina. Mar Ecol Prog Ser 289: 237-244.
65. Wu TY (2003) Reproductive biology of the blue shark, *Prionace glauca* in the Northwest Pacific. M. Sc. Thesis, National Taiwan Ocean University, Keelung, Taiwan.
66. Liu KM, Chen CT (1999) Demographic analysis of the scalloped hammerhead, *Sphyrna lewini*, in the northwestern Pacific. Fish Sci 65(2): 218-223.
67. Liu SZ (2002) Reproductive biology of the smooth hammerhead, *Sphyrna zygaena*, in the northeastern waters of Taiwan. M. Sc. Thesis, National Taiwan Ocean University, 84 pp.
68. Nammack MF, Musick JA, Colvocoresses JA (1985) Life history of the spiny dogfish off the Northeastern United States. Trans Amer Fish Soc 114: 367-376.
