# Supplementary material for: Estimating Finite Rate of Population Increase for Sharks Based on Vital Parameters
Source: PLoS One. 2015 Nov 17;10(11):e0143008. doi: 10.1371/journal.pone.0143008 (PMC4648575; doi:10.1371/journal.pone.0143008)
Supplement: S3 Table — (DOCX) [file pone.0143008.s003.docx]

S3-1 Table Finite population increase rate derived from demographic analysis for 62 stocks (38 species) of sharks.

| **Obs** | **Scientific name** | **M (yr^-1^)** | **T_m_ (yr)** | **T_max_ (yr)** | **R_0_** | **G (yr)** | **r (yr^-1^)** | **t_x2_ (yr)** | $\boldsymbol{\lambda}$ |
| --- | --- | --- | --- | --- | --- | --- | --- | --- | --- |
| 1 | *Alopias pelagicus* (NEP) | 0.1416 | 8.60 | 27.57 | 1.9929 | 14.3223 | 0.0481 | 14.3957 | 1.0493 |
| 2 | *A. superciliosus* (NET) | 0.1382 | 12.85 | 28.35 | 1.1620 | 17.9537 | 0.0084 | 82.8747 | 1.0084 |
| 3 | *A. vulpinus* (California) | 0.2061 | 5.00 | 17.94 | 2.7131 | 8.5412 | 0.1169 | 5.9315 | 1.1240 |
| 4 | *Carcharhinus acronotus* (NC) | 0.1537 | 3.50 | 25.11 | 8.2945 | 9.3294 | 0.2268 | 3.0567 | 1.2545 |
| 5 | *C. acronotus* (NWA) | 0.1938 | 4.50 | 19.25 | 2.5459 | 8.8095 | 0.1061 | 6.5343 | 1.1119 |
| 6 | *C. acronotus* (GM) | 0.2217 | 3.00 | 16.50 | 5.6101 | 6.4699 | 0.2666 | 2.6000 | 1.3055 |
| 7 | *C. amblyrhynchos* | 0.2928 | 7.00 | 12.00 | 0.5248 | 8.6881 | -0.0742 | 9.3413 | 0.9285 |
| 8 | *C. brachyurus* (SAF) | 0.0596 | 20.00 | 74.33 | 20.2561 | 34.2237 | 0.0879 | 7.8851 | 1.0919 |
| 9 | *C. brevipinna* (NET) | 0.2070 | 7.80 | 17.85 | 1.9470 | 11.0907 | 0.0601 | 11.5373 | 1.0619 |
| 10 | *C. brevipinna* (GM) | 0.1192 | 7.50 | 33.61 | 7.4124 | 14.7755 | 0.1356 | 5.1127 | 1.1452 |
| 11 | *C. falciformis* (Pacific) | 0.2008 | 6.50 | 18.48 | 2.6532 | 10.4652 | 0.0932 | 7.4341 | 1.0977 |
| 12 | *C. falciformis* (NET) | 0.1211 | 9.70 | 32.99 | 5.5553 | 16.3796 | 0.1047 | 6.6211 | 1.1104 |
| 13 | *C. falciformis* (NWGM) | 0.2119 | 8.00 | 17.38 | 1.5189 | 11.0525 | 0.0378 | 18.3281 | 1.0385 |
| 14 | *C. leucas* (SAF) | 0.1094 | 21.00 | 37.07 | 1.8170 | 26.7309 | 0.0223 | 31.0265 | 1.0226 |
| 15 | *C. leucas* (NGM) | 0.1111 | 18.00 | 36.42 | 2.2965 | 24.0792 | 0.0345 | 20.0753 | 1.0351 |
| 16 | *C. limbatus* (SAF) | 0.2700 | 7.00 | 13.17 | 0.5647 | 9.1832 | -0.0622 | 11.1384 | 0.9397 |
| 17 | *C. limbatus* (TB) | 0.2551 | 6.50 | 14.05 | 1.3397 | 9.4339 | 0.0310 | 22.3577 | 1.0315 |
| 18 | *C. longimanus* (SWEA) | 0.1449 | 6.50 | 26.87 | 4.4829 | 12.3627 | 0.1214 | 5.7117 | 1.1290 |
| 19 | *C. longimanus* (Pacific) | 0.1472 | 4.50 | 26.39 | 5.0737 | 10.50007 | 0.1547 | 4.4817 | 1.1673 |
| 20 | *C. obscurus* (NWP) | 0.0841 | 16.40 | 50.08 | 7.7272 | 26.4498 | 0.0773 | 8.9662 | 1.0804 |
| 21 | *C. obscurus* (NAU) | 0.0631 | 19.50 | 69.67 | 6.3057 | 33.2330 | 0.0554 | 12.5093 | 1.0570 |
| 22 | *C. obscurus* (NWA) | 0.0630 | 21.00 | 69.77 | 7.6625 | 34.1439 | 0.0596 | 11.6222 | 1.0615 |
| 23 | *C. plumbeus* (NET) | 0.2365 | 7.85 | 15.32 | 1.1884 | 10.5320 | 0.0164 | 42.2976 | 1.0165 |
| 24 | *C. plumbeus* (NWA1) | 0.0906 | 15.50 | 45.98 | 5.3420 | 24.5543 | 0.0682 | 10.1575 | 1.0706 |
| 25 | *C. plumbeus* (NWA3) | 0.1281 | 15.50 | 30.93 | 2.1000 | 20.9520 | 0.0354 | 19.5737 | 1.0360 |
| 26 | *C. plumbeus* (NWA4) | 0.0733 | 30.00 | 58.67 | 3.1453 | 39.4091 | 0.0291 | 23.8379 | 1.0295 |

M: natural mortality, T_m_: age at maturity, T_max_: maximum age, R_o_: net reproductive value per generation, G: generation time, r: intrinsic population growth rate, t_x2_: population doubling time, λ: finite rate of population increase.

S3-2 Table Finite population increase rate derived from demographic analysis for 62 stocks (38 species) of sharks.

| **Obs** | **Scientific name** | **M (yr^-1^)** | **T_m_ (yr)** | **T_max_ (yr)** | **R_0_** | **G (yr)** | **r (yr^-1^)** | **t_x2_ (yr)** | $\boldsymbol{\lambda}$ |
| --- | --- | --- | --- | --- | --- | --- | --- | --- | --- |
| 27 | *C. plumbeus* (WAU) | 0.0613 | 16.20 | 71.91 | 12.9039 | 30.9446 | 0.0826 | 8.3867 | 1.0862 |
| 28 | *C. porosus* (NB) | 0.1118 | 6.00 | 36.14 | 5.2836 | 13.5323 | 0.1230 | 5.6349 | 1.1309 |
| 29 | *C. signatus* (NEB) | 0.1623 | 10.00 | 23.58 | 3.7536 | 14.2337 | 0.0929 | 7.4589 | 1.0974 |
| 30 | *C. sorrah* (NAU) | 0.4740 | 2.50 | 6.91 | 0.8690 | 4.1335 | -0.0340 | 20.4126 | 0.9666 |
| 31 | *C. tilstoni* (NAU) | 0.1997 | 3.50 | 18.60 | 3.5744 | 7.8405 | 0.1625 | 4.2665 | 1.1764 |
| 32 | *Carcharodon carcharias* (SAF) | 0.0987 | 12.50 | 41.69 | 7.8270 | 21.0012 | 0.0980 | 7.0748 | 1.1029 |
| 33 | *Cetorhinus maximus* | 0.0867 | 5.00 | 48.39 | 7.6505 | 15.1151 | 0.1346 | 5.1490 | 1.1441 |
| 34 | *Chiloscyllitum plagiosum* (NT) | 0.3654 | 4.50 | 11.50 | 1.9897 | 6.8126 | 0.1010 | 6.8640 | 1.1063 |
| 35 | *Furgaleus macki* (SWA) | 0.3039 | 6.50 | 11.50 | 3.6231 | 8.6605 | 0.1486 | 4.6631 | 1.1603 |
| 36 | *Galeocerdo cuvier* (Hawaii) | 0.1987 | 5.00 | 18.71 | 14.9543 | 8.7471 | 0.3092 | 2.2414 | 1.3624 |
| 37 | *G. cuvier* (GM) | 0.2389 | 8.00 | 15.15 | 5.6393 | 10.5195 | 0.1644 | 4.2154 | 1.1787 |
| 38 | *G. cuvier* (Atlantic) | 0.1509 | 10.00 | 25.65 | 13.3669 | 14.7241 | 0.1761 | 3.9363 | 1.1925 |
| 39 | *Galerorhinus galeus* (NZ) | 0.1238 | 14.00 | 32.15 | 5.5999 | 19.7510 | 0.0872 | 7.9468 | 1.0911 |
| 40 | *G. galeus* (NZ) | 0.1097 | 8.00 | 36.94 | 14.8332 | 15.4652 | 0.1744 | 3.9748 | 1.1905 |
| 41 | *Isurus oxyrinchus* (NWP) | 0.1023 | 20.00 | 40.04 | 2.2025 | 26.6948 | 0.0296 | 23.4345 | 1.0300 |
| 42 | *I. oxyrinchus* (California) | 0.1074 | 7.50 | 37.86 | 6.0161 | 15.6692 | 0.1145 | 6.0526 | 1.1213 |
| 43 | *Lamna nasus* (NWA) | 0.0957 | 13.10 | 43.21 | 5.4469 | 22.2763 | 0.0761 | 9.1094 | 1.0791 |
| 44 | *Mustelus henlei* (CC) | 0.3828 | 3.00 | 10.98 | 1.9305 | 4.8475 | 0.1357 | 5.1083 | 1.1453 |
| 45 | *M. californicus* (CC) | 0.2210 | 2.10 | 16.56 | 4.3824 | 6.4776 | 0.2281 | 3.0387 | 1.2562 |
| 46 | *M. griseus* (NWT) | 0.1541 | 5.80 | 25.02 | 18.6716 | 11.1418 | 0.2627 | 2.6385 | 1.3004 |
| 47 | *M. manazo* (Taiwan) | 0.1768 | 2.00 | 21.38 | 5.3880 | 6.6445 | 0.2535 | 2.7346 | 1.2885 |
| 48 | *M. manazo* (Tokyo Bay) | 0.1601 | 4.50 | 23.96 | 4.3687 | 9.9115 | 0.1488 | 4.6594 | 1.1604 |
| 49 | *Negaprion brevirostris* (NEB) | 0.1102 | 9.70 | 36.75 | 8.3527 | 17.2419 | 0.1231 | 5.6305 | 1.1310 |
| 50 | *Notorynchus cepedianus* (NEP) | 0.1397 | 15.95 | 28.00 | 13.5559 | 20.1440 | 0.1294 | 5.3562 | 1.1382 |
| 51 | *Prionace glauca* (NWP) | 0.2134 | 4.20 | 17.24 | 12.3219 | 8.4603 | 0.2968 | 2.3351 | 1.3456 |
| 52 | *P. glauca* (NEP) | 0.2800 | 6.50 | 12.36 | 10.1623 | 8.9472 | 0.2592 | 2.6747 | 1.2958 |

M: natural mortality, T_m_: age at maturity, T_max_: maximum age, R_o_: net reproductive value per generation, G: generation time, r: intrinsic population growth rate, t_x2_: population doubling time, λ: finite rate of population increase.

S3-3 Table Finite population increase rate derived from demographic analysis for 62 stocks (38 species) of sharks.

| **Obs** | **Scientific name** | **M (yr^-1^)** | **T_m_ (yr)** | **T_max_ (yr)** | **R_0_** | **G (yr)** | **r (yr^-1^)** | **t_x2_ (yr)** | $\boldsymbol{\lambda}$ |
| --- | --- | --- | --- | --- | --- | --- | --- | --- | --- |
| 53 | *Sphyrna lewini* (NET) | 0.3012 | 4.70 | 11.62 | 5.0076 | 7.0555 | 0.2283 | 3.0358 | 1.2562 |
| 54 | *S. lewini* (NWGM) | 0.1050 | 15.00 | 38.84 | 14.4427 | 22.0795 | 0.1209 | 5.7316 | 1.1286 |
| 55 | *S. zygaena* (NET) | 0.1504 | 11.00 | 25.73 | 9.3396 | 15.5758 | 0.1434 | 4.8322 | 1.1542 |
| 56 | *Squalus acanthias* (SEBS) | 0.2172 | 5.00 | 16.89 | 2.2223 | 8.3011 | 0.0962 | 7.2056 | 1.1010 |
| 57 | *S. acanthias* (NWA) | 0.1519 | 12.10 | 25.44 | 0.9538 | 17.2002 | -0.0027 | 252.2765 | 0.9973 |
| 58 | *S. acanthias* (NEP) | 0.0581 | 29.00 | 76.51 | 3.6629 | 42.6985 | 0.0304 | 22.7972 | 1.0309 |
| 59 | *S. acanthias* (Canada) | 0.0554 | 23.00 | 80.81 | 6.0747 | 38.2221 | 0.0472 | 14.6850 | 1.0483 |
| 60 | *S. blainville* (Italy) | 0.1398 | 5.10 | 27.99 | 2.6506 | 11.7035 | 0.0833 | 8.3220 | 1.0869 |
| 61 | *Sphyna tiburo* (NWF) | 0.2314 | 4.00 | 15.71 | 10.0310 | 7.1656 | 0.3218 | 2.1542 | 1.3796 |
| 62 | *Scoliodon laticaudus* (India) | 0.4703 | 1.50 | 8.96 | 3.8119 | 3.4752 | 0.3850 | 1.8002 | 1.4697 |

M: natural mortality, T_m_: age at maturity, T_max_: maximum age, R_o_: net reproductive value per generation, G: generation time, r: intrinsic population growth rate, t_x2_: population doubling time, λ: finite rate of population increase.
